# The Mgv1–Rlm1 axis orchestrates SAGA and SWI/SNF complexes at target promoters

**DOI:** 10.1093/nar/gkaf653

**Published:** 2025-07-12

**Authors:** Chaoyun Xu, Yueqi Zhang, Chengqi Zhang, Li Chen, Yanni Yin, Yun Chen, Zunyong Liu, Zhonghua Ma

**Affiliations:** Institute of Biotechnology, National Key Laboratory of Rice Biological Breeding, Key Laboratory of Molecular Biology of Crop Pathogens and Insects, Zhejiang University, Hangzhou 310058, China; Zhongshan Biological Breeding Laboratory, CIMMYT–JAAS Joint Center for Wheat Diseases, Key Laboratory of Germplasm Innovation in Downstream of Huaihe River (Nanjing), Ministry of Agriculture and Rural Affairs, Jiangsu Academy of Agricultural Sciences, Nanjing 210014, China; Institute of Biotechnology, National Key Laboratory of Rice Biological Breeding, Key Laboratory of Molecular Biology of Crop Pathogens and Insects, Zhejiang University, Hangzhou 310058, China; Department of Plant Pathology, School of Plant Protection, Anhui Agricultural University, Hefei 230036, China; Department of Plant Pathology, School of Plant Protection, Anhui Agricultural University, Hefei 230036, China; Institute of Biotechnology, National Key Laboratory of Rice Biological Breeding, Key Laboratory of Molecular Biology of Crop Pathogens and Insects, Zhejiang University, Hangzhou 310058, China; Institute of Biotechnology, National Key Laboratory of Rice Biological Breeding, Key Laboratory of Molecular Biology of Crop Pathogens and Insects, Zhejiang University, Hangzhou 310058, China; Institute of Biotechnology, National Key Laboratory of Rice Biological Breeding, Key Laboratory of Molecular Biology of Crop Pathogens and Insects, Zhejiang University, Hangzhou 310058, China; Institute of Biotechnology, National Key Laboratory of Rice Biological Breeding, Key Laboratory of Molecular Biology of Crop Pathogens and Insects, Zhejiang University, Hangzhou 310058, China; Yazhouwan National Laboratory, Sanya 572025, China

## Abstract

The SWI/sucrose non-fermentable (SWI/SNF)-facilitated removal of nucleosomes and Spt-Ada-Gcn5 acetyltransferase (SAGA) complex-mediated histone acetylation are crucial for the activation of transcription initiation. However, the mechanism by which these two complexes coordinate to regulate gene expression involved in cell wall remodeling during infection process or in response to external stimuli remains largely unknown in plant pathogenic fungi. Here, we demonstrate that the cell wall integrity (CWI) pathway is activated under toxin (deoxynivalenol)-inducing conditions in the phytopathogenic fungus *Fusarium graminearum*. This treatment results in the phosphorylation and nuclear translocation of the mitogen-activated protein kinase FgMgv1 in the CWI signaling pathway. Once in the nucleus, the activated FgMgv1 phosphorylates the downstream transcription factor FgRlm1, which binds to a 12- or 14-bp *cis*-element in the promoters of target genes. Notably, FgMgv1 forms a polymer and interacts with FgRlm1 via its kinase domain. Crucially, this polymerization enables FgMgv1 to recruit both the SWI/SNF and SAGA complexes simultaneously through its C-terminal domain at the target promoters. This coordinated action among FgMgv1, FgRlm1, SWI/SNF, and SAGA ultimately facilitates the transcriptional activation of target genes. Collectively, these findings illuminate a regulatory framework in which Mgv1–Rlm1 axis serves as a key regulatory hub, integrating CWI signals with epigenetic modifications to ensure transcriptional responsiveness to external stimuli.

## Graphical Abstract

**Graphical Abstract:**
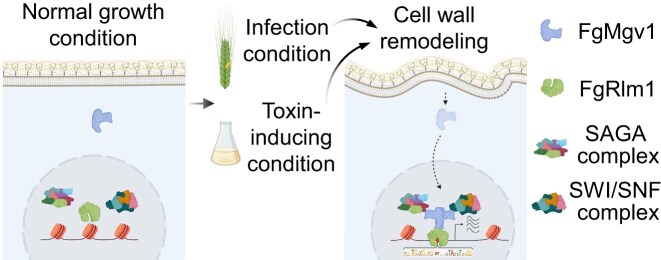

## Introduction

During gene transcription process, nucleosome removal at the target genes in the high complexity of DNA packaged into chromatin is an important step [1]. The SWI/sucrose non-fermentable (SWI/SNF) complex is large chromatin remodeling machine that can move or eject nucleosomes, promoting transcription and other nuclear processes [2–4]. Due to its inability to directly bind specific DNA sequences, the SWI/SNF complex relies on interactions with gene-specific activators for target recruitment [5–8]. In addition, chromatin modulation through histone modifications, such as methylation and acetylation, plays a vital role in regulating DNA accessibility during transcription [9]. The SAGA (Spt-Ada-Gcn5 acetyltransferase) complex is a highly conserved co-activator that modulates histone acetylation, thereby promoting an open chromatin conformation conducive to gene expression under stress conditions [10]. This histone modification has been found to be responsible for an open chromatin conformation and gene expression under stress conditions [11]. Moreover, the interplay between SAGA and SWI/SNF complexes is essential for an effective transcriptional response [12–14], especially under cell wall stress conditions, as evidenced by the co-regulation of ∼65% of induced genes in genome-wide expression analyses in the budding yeast [7, 15–18]. Thus, it would be interesting to explore the mechanisms by which these two complexes coordinate to regulate gene expression in response to external stimuli.

The fungal conversed cell wall integrity (CWI) signaling pathway is a specialized mitogen-activated protein kinase (MAPK) signaling pathway that plays a vital role in maintaining cell wall stability under stress conditions [19–21]. In *Saccharomyces cerevisiae*, the pathway begins with cell-surface sensors, including Wsc1, Wsc2, Wsc3, Mid2, and Mtl1. These sensors activate the guanine nucleotide exchange factors Rom1 and Rom2, which then promote nucleotide exchange on the small GTPase Rho1. Activated Rho1 initiates the protein kinase C (Pkc1), which subsequently triggers a MAPK cascade involving Bck1 (MAPKKK), Mkk1/Mkk2 (MAPKKs), and Slt2/Mpk1/Mgv1 (MAPK) [22, 23]. Upon activation, MAPK Slt2 translocates to the nucleus, where it activates transcription factors such as Rlm1 and the Swi4/Swi6 complex (SBF) [24], and Rlm1 is primarily responsible for the transcriptional activation of genes involved in cell wall stress response [25, 26]. In addition to phosphorylating transcription factors, Slt2 has been shown to activate the transcription of certain CWI responsive genes independently of its catalytic mechanism [27]. This suggests that Slt2 homologues may have unidentified roles in regulating gene transcription for CWI in fungal responses to environmental stimuli. In the yeast, cell wall stress conditions activated Slt2 to phosphorylates Rlm1, which interacts with SWI/SNF and probably the SAGA complexes to direct both to the promoters of CWI responsive genes [15, 25, 28, 29].

Although the fundamental biophysical design of the cell wall is conserved among diverse fungi, each fungal species may possess a unique chemical composition [30]. The architectural and mechanical properties of fungal cell walls are adaptable, accommodating the requirements of various commensal, symbiotic, and pathogenic fungal lifestyles [30]. For example, phytopathogenic fungi can develop appressoria that generate high pressures through the infection peg, allowing them to breach the tough surfaces of plants and facilitate invasion and pathogenesis [30–32]. Thus, it would be interesting to investigate how Slt2 homologues, also named Mgv1 in *Fusarium graminearum* (hereafter named *Fg*), precisely regulate cell wall remodeling and morphological changes during infection of phytopathogenic fungi.

*Fg* is a major pathogen responsible for *Fusarium* head blight (FHB), a disease that causes severe economic losses in small grain cereal crops worldwide [33]. Recently, there have been heavy epidemics of FHB, leading to significant reductions in yield, substantial economic damage, and increased concerns regarding mycotoxin contamination in food and feed. The primary mycotoxin of concern is deoxynivalenol (DON), a type B trichothecene, whose biosynthetic enzymes are encoded by 15 *TRI* genes [34]. Previous studies have demonstrated that the transcription of the *TRI* genes and histone acetylation are rapidly induced when putrescine is used as the sole nitrogen source in toxin biosynthesis-inducing (TBI) medium [33, 35], which provides an ideal model for investigating the transcription regulatory mechanisms of fungal responses to environmental stimuli.

In current study, we found that the CWI pathway of *Fg* is activated under TBI conditions. More importantly, the FgMgv1 (Slt2 homologue)–FgRlm1 axis coordinates the SWI/SNF and SAGA complexes to regulate the transcription of target genes involved in the CWI under TBI conditions. Mechanistically, under TBI conditions, the activated FgMgv1 phosphorylates its downstream transcription factor FgRlm1, which binds to a 12- or 14-bp *cis*-element in the target gene promoters. In nucleus, FgMgv1 forms a polymer and interacts with FgRlm1 via its MAPK kinase domain. The polymer of FgMgv1 further recruits both the SWI/SNF and SAGA complexes through its C-terminal domain at the target promoters. The orchestration among FgMgv1, FgRlm1, SWI/SNF, and SAGA complexes ultimately leads to the activation of transcription of the target genes for CWI, which further regulates DON biosynthesis and virulence of *Fg*. This study uncovers a comprehensive network in which FgMgv1 functions as a scaffold protein, bridging CWI pathway with epigenetic modifications to regulate target gene transcription.

## Materials and methods

### Strain construction

The wild-type strain PH-1 (NRRL 31084) of *Fg* was used as a parental strain for transformation experiments. To obtain protoplasts, fresh mycelia were treated with driselase (D9515; Sigma), lysozyme (RM1027; RYON, Shanghai, China), and cellulose (RM1030; RYON). The fusion DNA fragments for target gene deletion were constructed by using double-joint PCR (polymerase chain reaction) method [36]. Each open reading frame (ORF) was replaced with HPH (hygromycin resistance) gene, and then, deletion mutants were identified by PCR with specific primers. For complementation, the corresponding ORF was fused to the geneticin resistant gene and a fluorescence protein gene (e.g. GFP), and the resulting DNA fragment was introduced into the corresponding deletion mutant. The primers used to amplify the flanking sequences for each gene are listed in Supplementary Table S1.

### Cell wall integrity determination

To determine the sensitivity of mycelia to cell wall-degrading enzymes, mycelia were grown in complete medium (CM) at 25°C in a shaker at 180 rpm or in TBI medium at 28°C in a shaker at 150 rpm. The collected mycelia were treated with driselase, lysozyme, and cellulose at 30°C for 30 min in a shaker at 85 rpm. Then, the mycelia were observed under the Zeiss LSM780 confocal microscope (Göttingen, Niedersachsen, Germany) for detection of abandoned protoplasts. Three technical replicates were used for each strain and each experiment was repeated three times independently.

To determine the sensitivity of mycelia to cell wall stress agent sodium dodecyl sulfate (SDS) and Congo red (CR), mycelial plugs of each strain were inoculated on minimal medium plates supplemented with 0.02% SDS or CR. After incubation at 25°C for 3 days in the dark, the diameter of each colony was measured for calculation of mycelial growth inhibition rate. For non-round colonies, three diameters were measured and averaged. Three technical replicates were used for each strain and each experiment was repeated three times independently.

### RNA-seq assay and real time quantitative PCR (RT-qPCR)

For RNA-seq assay, the wild-type strain PH-1 and mutant strains were incubated in TBI at 28°C in a shaker at 150 rpm for 24 h. Total RNA was isolated from fresh mycelia frozen in liquid nitrogen with the TRIzol Reagent (TaKaRa Biotechnology Co. Ltd, Dalian, China) and shipped to Novogene Co. Ltd (Beijing, China) for library construction. Sequencing libraries were generated using NEBNext Ultra RNA Library Prep Kit for Illumina (E7530L, NEB, USA) following manufacturer’s recommendations and index codes were added to attribute sequences to each sample. Briefly, mRNA was purified from total RNA using poly-T oligo-attached magnetic beads. AMPure XP system (Beverly, USA) wad used to purify the library fragments. Library quality was assessed on the Agilent 5400 system (Agilent, USA) and quantified by quantitative PCR (qPCR) (1.5 nM). High-throughput sequencing was performed on Hiseq-PE150. The original fluorescence image files obtained from Illumina platform are transformed to short reads (raw data) by base calling and these short reads are recorded in FASTQ format, which contains sequence information and corresponding sequencing quality information. Fastp (version 0.23.1) was used to perform basic statistics on the quality of the raw reads. The resulting clean RNA-seq reads were mapped onto the reference genome of *Fg* with HISAT2 (v.2.1.0) [37]. The numbers of reads mapped to each gene were counted with the HTSeq-count [38]. Differential expression analysis for the tested strains was performed with DESEQ2 R package [39]. The threshold values with fold change of ≥2 and *P*-value of ≤.05 were used to identify differentially expressed genes. Each experiment was repeated three times independently.

For RT-qPCR, RNA Extraction Kit (9109; TaKaRa Biotechnology Co. Ltd, Beijing, China) was used to purify total RNA of each strain isolated from fresh mycelia frozen in liquid nitrogen. The HiScriptII Q RT SuperMix for qPCR kit was used for genomic DNA digestion and complementary DNA (cDNA) synthesis. SYBR qPCR Master Mix Kit (TaKaRa Biotechnology Co. Ltd) was used to examine relative expressions of target genes with the primers listed in Supplementary Table S1. *FgACTIN* gene was used as the internal control. Three technical replicates were used for each strain, and each experiment was repeated three times independently.

### Co-immunoprecipitation (Co-IP) assay

DNA fragments of target genes fused with different marker genes (e.g. GFP or 3× Flag) were transformed into pairs into PH-1. Total protein of each strain containing a pair of fusion constructs was extracted with extraction buffer (50 mM Tris–HCl, pH 7.5, 100 mM NaCl, 5 mM EDTA, 1% Triton X-100) containing 10 μl of protein inhibitor cocktail (Sangon Biotech Co. Ltd, Shanghai, China). After leaving on ice for 30 min, the lysates were centrifuged at 12 000 × *g* for 10 min at 4°C and then the supernatants were incubated with the anti-GFP agarose (ChromoTek, Martinsried, Germany). Western blot assays were used to analyze proteins eluted from agarose. Each experiment was repeated three times independently.

### Western blotting assays

Protein extraction was performed as described above. The resulting proteins were separated by 10% sodium dodecyl sulfate–polyacrylamide gel electrophoresis and transferred to an Immobilon-P transfer membrane (Millipore, Billerica, MA, USA). Western blotting was conducted with the mouse monoclonal anti-Flag (A9044; Sigma) and the rabbit polyclonal anti-GFP (ab32146; Abcam, Cambridge, UK) antibody at 1:5000 dilution. The mouse monoclonal anti-GAPDH antibody (EM1101; HuaBio Co. Ltd, Hangzhou, China) at 1:5000 dilution was used as a reference. The goat polyclonal anti-rabbit IgG-HRP (HA1001; HuaBio Co. Ltd) or the goat polyclonal anti-mouse IgG-HRP (HA1006; HuaBio Co. Ltd) was used as the secondary antibody. All the blots with chemiluminescence were imaged by Image Quant LAS4000 mini (GE Healthcare, Chicago, IL, USA). Each experiment was repeated three times independently.

### Yeast two-hybrid （Y2H） assays

The cDNA of *Fg* was used to clone the coding sequence of each tested gene with primers listed in Supplementary Table S1 to construct plasmids for Y2H analyses. The cDNA fragment was inserted into the yeast GAL4-binding domain vector pGBKT7 and GAL4-activation domain vector pGADT7 (Clontech, Mountain View, CA, USA), respectively. The pairs of Y2H plasmids were transformed into the *S. cerevisiae* strain Y2H Gold with the lithium acetate/single stranded DNA/polyethylene glycol transformation protocol. The pair of pGBKT7-53 and pGADT7-T plasmids was used as positive control, and the pair of pGBKT7-Lam and pGADT7-T plasmids was used as negative control. After culturing on minimal synthetic defined (SD) medium lacking Leu and Trp at 30°C for 3 days, the transformants were transferred to SD medium lacking His, Leu, Trp containing 2.5 mM 3-aminotriazole (3-AT, A8056, Sigma, MO, USA) to assess protein–protein interaction. Each experiment was repeated three times independently.

### Bimolecular fluorescence complementation (BiFC) assay

DNA fragments of target genes fused with YFP^N^ or YFP^C^ fragment were introduced into the wild-type strain. Each strain was cultured in CM at 25°C for 16 h or in TBI at 28°C for 24 h. The Zeiss LSM780 confocal microscopy (Göttingen, Niedersachsen, Germany) was used to observe fluorescent intensity and localization of tagged proteins. Each experiment was repeated three times independently.

### Chromatin immunoprecipitation sequencing (ChIP-seq) and ChIP–qPCR

ChIP-seq was performed according to a published protocol with minor modifications [40]. Briefly, fresh mycelia of each strain cultured in TBI medium at 28°C for 24 h were cross-linked with 1% formaldehyde for 15 min and then stopped with 125 mM glycine. The resulting mycelia were resuspended in the lysis buffer [250 mM HEPES, pH 7.5, 150 mM NaCl, 1 mM EDTA, 1% Triton, 0.1% deoxycholate, 10 mM dithiothreitol (DTT)] and protease inhibitor (Sangon Co., Shanghai, China). The Bioruptor Plus Diagenode UCD-300 was used to shear DNAs into 200–500 bp fragments with 30 s on and 30 s off. After centrifugation, supernatant of each sample was mixed with the rabbit polyclonal anti-GFP antibody (ab290; Abcam, Cambridge, UK) at 1:500 dilution, the rabbit polyclonal anti-acetyl-histone H3K27 (a7253; ABclonal Co., Wuhan, China), or the rabbit polyclonal anti-acetyl-histone H3K18 (a7257; ABclonal Co., Wuhan, China) together with the protein A agarose beads (sc-2001; Santa Cruz, CA, USA), respectively. After washing orderly by low-salt wash buffer (one time; 150 mM NaCl, 20 mM Tris–HCl, pH 8.0, 0.2% SDS, 0.5% Triton X-100, 2 mM EDTA), high-salt wash buffer (one time; 500 mM NaCl, 20 mM Tris–HCl, pH 8.0, 0.2% SDS, 0.5% Triton X-100, 2 mM EDTA), LiCl wash buffer, TE buffer (two times; 100 mM Tris–HCl, pH 8.0, 10 mM EDTA), DNA samples were eluted from beads with elution buffer (1% SDS, 0.1 M NaHCO_3_) and then immunoprecipitated by ethanol after washing, eluting, reversing the cross-linking, and digesting with proteinase K. The purified DNA was used for ChIP-seq library preparation. The library was constructed by the commercial service of Genergy Biotechnology Co. Ltd (Shanghai, China). Subsequently, pair-end sequencing of sample was performed on Illumina platform (Illumina, CA, USA). Library quality was assessed on the Agilent Bioanalyzer 2100 system.

Raw data of FASTQ format were first processed using Fastp (v. 0.22.0) software. Clean data were obtained by removing reads containing adapter, reads containing poly-N, and low-quality reads from raw data. At the same time, Q20, Q30, and GC content of the clean data were calculated. All the downstream analyses were based on the clean data with high quality. Index of the reference genome was built using BWA (v. 0.7.17) and clean reads were aligned to the reference genome using BWA mem (v. 0.7.17). For a specific ChIP-seq binding site, individual reads are mapped to the plus or minus strand and present significant enrich. After mapping reads to the reference genome, we used the MACS2 (version 2.2.7.1) software to identify regions of IP enrichment over background. A *q*-value threshold of 0.05 was used for all datasets. After peak calling, the distribution of chromosome distribution, peak width, fold enrichment, significant level, and peak summit number per peak were all displayed. Each experiment was repeated twice independently.

The multiple EM for motif elicitation (MEME) program MEME-ChIP was used to analyze 500-bp sequences around the peak summits [41], identifying the binding *cis*-element of FgRlm1.

For ChIP quantitative real-time PCR analysis, SYBR Green I fluorescent detection dye was used in the PCR assays. Three technical replicates were used for each strain and each experiment was repeated three times independently.

### Electrophoretic mobility shift assay (EMSA)

The fragment of FgRlm1 coding sequence was inserted into home-modified pET28b vector with Strep-tag at their C-terminus for prokaryotic expression and protein purification of FgRlm1. The plasmid was transformed into the *Escherichia coli* strain BL21. The strain was cultured in LB medium overnight at 37°C. The cultures were diluted 1:50 in fresh LB medium and grown at 37°C until OD_600_ of 0.5–0.6. After adding 1 mM isopropyl-β-d-thiogalactopyranoside, cultures were induced overnight at 16°C. After sonication step, the Strep-tagged FgRlm1 was purified by a Strep-tag affinity chromatography column and washed with lysis buffer (25 mM Tris–HCl, pH 7.5, 500 mM NaCl, 1 mM DTT, 1% Triton X-100, protease inhibitors, 1 mM PMSF) for four times. Purified proteins were eluted in lysis buffer containing 10 mM desthiobiotin.

The DNA fragments of *cis*-element of FgRlm1 with three repeats were labeled with biotin by Sangon Biotech Co. (Shanghai, China). The LightShift™ Chemiluminescent EMSA Kit 20148 (Thermo Fisher Scientific Inc., MA, USA) was used for EMSA. Briefly, the mixtures of FgRlm1-Strep protein and biotin-labeled probe were incubated for 10 min at room temperature, and reactions were then electrophoresed on 6% polyacrylamide gel in 0.5× TBE and transferred to a positively charged nylon membrane (INYC00010; Millipore, Boston, USA). Signals were detected using the chemiluminescent substrate in the kit according to the manufacturer’s instructions. Each experiment was repeated three times independently.

### The micrococcal nuclease (MNase)–qPCR assays

MNase assay was conducted according to a previously reported protocol [42]. Briefly, mycelia of each strain were mixed with lysis buffer [250 mM sucrose, 25% (v/v) glycerol, 2 mM MgCl_2_, 20 mM KCl, 20 mM Tris–HCl (pH 7.5), 5 mM DTT]. The mixture was incubated on ice for 5 min and then filtered through cheesecloth. After centrifugation at 15 000 × *g* for 15 min at 4°C, the pellets were washed with NEB1 (Nuclei Extraction Buffer 1) buffer [20 mM Tris–HCl (pH 7.5), 0.2% (w/v) Triton X-100, 25% (v/v) glycerol, 2.5 mM MgCl_2_] for five times. The resulting pellets were resuspended with NEB2 buffer [20 mM Tris–HCl (pH 7.5), 0.5% (w/v) Triton X-100, 250 mM sucrose, 10 mM MgCl_2_, 5 mM β-mercaptoethanol] and then layered onto NEB3 buffer [20 mM Tris–HCl (pH 7.5), 0.5% (w/v) Triton X-100, 1.7 M sucrose, 10 mM MgCl_2_, 5 mM β-mercaptoethanol]. After centrifugation at 16 000 × *g* for 45 min at 4°C, the final nuclei pellet was suspended in MNase reaction buffer [20 mM Tris–HCl (pH 8.0), 5 mM NaCl, 2.5 mM CaCl_2_]. An equal portion of nuclei isolation (160 μl) of each sample was mixed with 320 μl with MNase reaction buffer and treated with 4 μl MNase enzyme (Takara, Beijing, China) to conduct MNase digestion. The same samples without MNase enzyme treatment were used as the undigested control. All samples were incubated at 37°C for 8 min and added with 50 μl of stop buffer (50 μl of 10% SDS and 40 μg proteinase K) at 60°C for 1 h. After treating with 1 U RNase (10 μg/μl) at 37°C for 1 h, the resulting samples were stored at 4°C overnight. DNAs from each sample were extracted by using the phenol–chloroform–isoamyl alcohol method and resuspended in 50 μl water. The resulting DNA samples were used for qPCR with multiple pairs of primers spanning the tested region (Supplementary Table S1). Three technical replicates were used for each strain and each experiment was repeated three times independently.

### Analysis of DON production

Each tested strain was cultured in TBI at 28°C for 7 days in a shaker at 150 rpm. The resulting supernatant was collected for DON extraction, and the mycelia were dried and weighed. DON production of each sample was quantified by DON Quantification Kit Wis008 (Wise Science, Zhenjiang, China). Three technical replicates were used for each strain and each experiment was repeated three times independently.

### Pathogenicity determination

Given that mutant of the SAGA complex was defective in conidiation, we used fresh mycelia for pathogenicity. Briefly, 0.1 mg of fresh mycelia of each strain was inoculated to the middle spikelet of flowering wheat heads. Each strain was inoculated in 20 individual wheat heads, and the images were taken at 15 days after inoculation. To determine pathogenicity on corn silks and wheat coleoptiles, 5-mm mycelial plugs of each strain taken from colony edge were inoculated on corn silks and wheat coleoptiles. Each strain was inoculated in 15 individual wheat coleoptiles or corn silk samples, and images were taken at 4 days after inoculation. Each experiment was repeated three times independently.

## Results

### Cell wall integrity pathway is activated in *Fg* under toxin-inducing conditions

**Figure 1.**
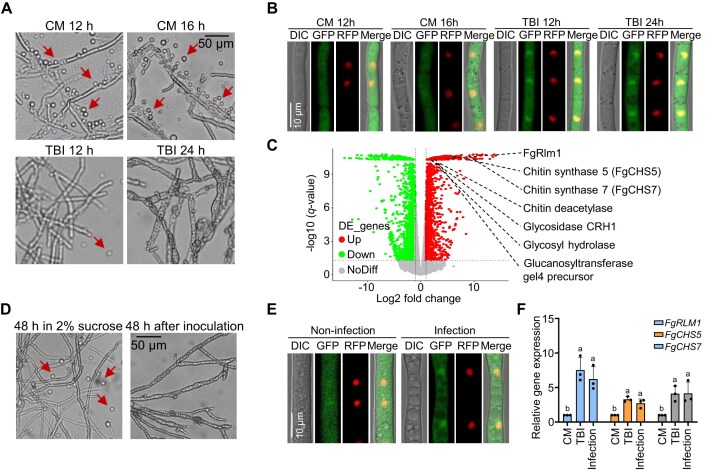
Cell wall integrity pathway is activated under toxin-inducing and infection conditions. (**A**) *Fg* under toxin-inducing conditions is more resistant to cell wall lytic enzymes. Wild-type PH-1 grown in the CM or TBI medium at indicated time points were treated with cellulase, lysozyme, and driselase for 30 min at 30°C. The mycelia of *Fg* grown in CM was more sensitive to cell wall lytic enzymes compared to those grown in TBI. The arrows indicate the protoplast released by cell wall lytic enzymes. Bar = 50 μm. (**B**) FgMgv1 is translocated into the cell nucleus under TBI conditions. ΔFgMgv1::GFP-FgMgv1::H1-RFP grown in the CM and TBI conditions for indicated time was subject to confocal analysis. Bar = 10 μm. (**C**) CWI-related genes are upregulated under TBI conditions. Scatter plot showing comparisons in expression levels from RNA-seq assays of the wild-type strain cultured in CM medium for 16 h and in TBI medium for 24 h. DE_genes indicates the differentially expressed genes. Up- and downregulated genes are highlighted in red and green, respectively. The remaining genes are shown in gray. Genes with a fold change ≥2 and a *P*-value ≤.05 were considered significantly differentially expressed. The CWI-related genes are indicated in the figure. (**D**) *Fg* under infection conditions is more resistant to cell wall lytic enzymes. Mycelia from spore germination grown in 2% sucrose or inoculated on the wheat leaves 48 h post-inoculation (hpi) were treated with cellulase, lysozyme, and driselase for 30 min at 30°C. Bar = 50 μm. (**E**) FgMgv1 is translocated into the cell nucleus under infection conditions. ΔFgMgv1::GFP-FgMgv1::H1-RFP grown in 2% sucrose or inoculated on the wheat leaves 48 hpi was subject to confocal analysis. Bar = 10 μm. (**F**) CWI-related genes are upregulated in wild-type PH-1 under toxin-inducing and infection conditions. RNA samples were extracted from fresh mycelia grown in CM medium for 16 h, in TBI medium for 24 h, and inoculated on the wheat leaves after 48 h. Line bar in each column denotes standard deviation of three repeated experiments. Different letters indicate significant differences based on ANOVA followed by Fisher’s least significant difference (LSD) test (*P*= .05).

*Fg* undergoes dramatic hyphal morphology changes after incubation in TBI for 24 h (Fig. 1A) [33], suggesting a potential activation of the CWI pathway in TBI condition. To test this, *Fg* mycelia were grown in CM for vegetative growth and in TBI medium that induces DON biosynthesis, yielding comparable biomass under both conditions (Supplementary Fig. S1A). After treatment with cell wall-degrading enzymes, the CM-grown mycelia were easily digested, generating more protoplasts than those grown in TBI medium, indicating enhanced cell wall robustness under TBI conditions (Fig. 1A and Supplementary Fig. S2). Since translocation of Mgv1 from the cytoplasm to the nucleus indicates activation of the CWI pathway [43, 44], the subcellular localization of FgMgv1 was examined in both CM and TBI. Consistent with the observed resistance to cell wall-degrading enzymes, the TBI conditions promoted the nuclear import of GFP-FgMgv1 with H1 (histone1)-RFP as the nuclear marker (Fig. 1B). However, TBI medium lacking a nitrogen source—which does not induce toxin production—as well as CM medium supplemented with either a nitrogen source or exogenous DON did not alter the subcellular localization of FgMgv1 and FgRlm1 (Supplementary Fig. S3A–C). In addition, RNA-seq analysis revealed a significant upregulation of cell wall biosynthesis-related genes, including those involved in chitin synthesis, glycosidase activity, and chitin deacetylase activity, under TBI compared to CM conditions (Fig. 1C and Supplementary Fig. S4A–C). In line with that in TBI, inoculation on wheat also resulted in increased mycelial resistance to cell wall digestion enzymes, nuclear translocation of FgMgv1, and upregulation of *FgCHS* genes (Fig. 1D–F). Further, the FgRlm1-GFP were observed in infection cushion structure (Supplementary Fig. S5). These findings collectively indicate that the CWI pathway is activated in *Fg* under TBI conditions and during infection process.

### FgMgv1 interacts with the downstream transcription factor FgRlm1 via its MAPK domain

**Figure 2.**
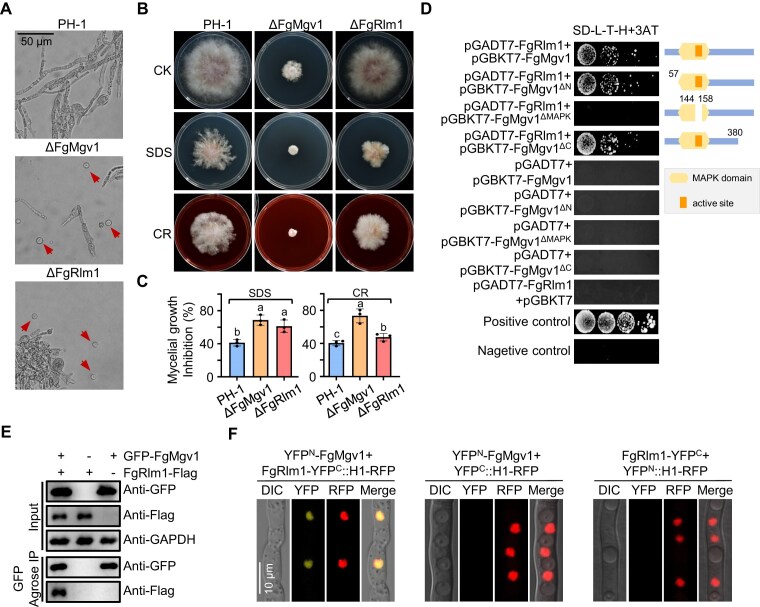
FgMgv1 interacts with the downstream transcription factor FgRlm1 via its MAPK domain. (**A**) Deletion of FgMgv1 and FgRlm1 leads to more sensitivity to cell wall lytic enzymes compared to the wild type. The wild-type PH-1, ΔFgMgv1, and ΔFgRlm1 grown in the TBI medium were treated with cellulase, lysozyme, and driselase for 30 min at 30°C. The arrows indicate the protoplast released by cell wall lytic enzymes. Bar = 50 μm. (B, C) ΔFgMgv1 and ΔFgRlm1 are more sensitive to SDS and CR compared with the wild type. Colony morphology (**B**) and mycelial inhibition (**C**) were examined after growth on minimal medium (MM; CK) with or without 0.02% SDS or 0.02% CR for 3 days. Different letters indicate significant differences based on ANOVA followed by Fisher’s LSD test (*P* = .05). (**D**) FgMgv1 interacts with FgRlm1 in a manner dependent on the active site of its MAPK kinase domain, as demonstrated by the Y2H assay. Interactions were determined by monitoring the growth on SD medium lacking leucine (L), tryptophan (T), and histidine (H), but supplemented with 3-AT (SD-L-T-H + 3-AT) of yeast cells bearing a pair of vectors as indicated. pGBKT7-53 and pGADT7-T were used as positive control, and pGBKT7-Lam and pGADT7-T were used as negative control. (**E**) FgMgv1 interacts with FgRlm1 in the co-immunoprecipitation (co-IP) assay. Protein samples were extracted from fresh mycelia of each strain incubated in TBI medium for 24 h, with protein loading amounts verified using the anti-GAPDH. (**F**) FgMgv1 is associated with FgRlm1 in nucleus in BiFC assay. The strains containing YFP^N^-FgMgv1, FgRlm1-YFP^C^, and H1-RFP were incubated in TBI medium for 24 h. Bar = 10 μm.

The transcription factor Rlm1 is a downstream transcription factor of Mgv1 in the CWI pathway [45]. Consistent with this finding, FgRlm1 was significantly upregulated under the FgMgv1 activation conditions including TBI and infection process (Fig. 1C and Supplementary Fig. S4), indicating FgMgv1 may also regulate expression level of FgRlm1. To elucidate its function, ΔFgRlm1 and ΔFgMgv1 mutants were generated using marker-assisted homologous recombination (Supplementary Fig. S6A and B). Cell stress sensitivity assays revealed that, like ΔFgMgv1, ΔFgRlm1 exhibited high sensitivity to cell wall-degrading enzymes as compared to the wild-type PH-1 cultured in TBI for 24 h (Fig. 2A). Additionally, these mutants showed increased sensitivity to cell wall-damaging agents CR and SDS (Fig. 2B and C). Y2H assay demonstrated that FgMgv1 directly interacts with FgRlm1 and this interaction is dependent on the MAPK domain of FgMgv1 (Fig. 2D and Supplementary Fig. S7). In addition, FgMgv1 was co-immunoprecipitated with FgRlm1 in TBI conditions, but not under CM condition (Fig. 2E and Supplementary Fig. S8A). Furthermore, BiFC assay indicated that FgMgv1 interacts with FgRlm1 in the nucleus under TBI conditions but not under CM condition (Fig. 2F and Supplementary Fig. S8B). These results collectively indicate that FgMgv1 interacts with the transcription factor FgRlm1 through its MAPK domain under TBI conditions.

### FgRlm1 regulates CWI via recruiting FgMgv1 to the promoters of target genes

**Figure 3.**
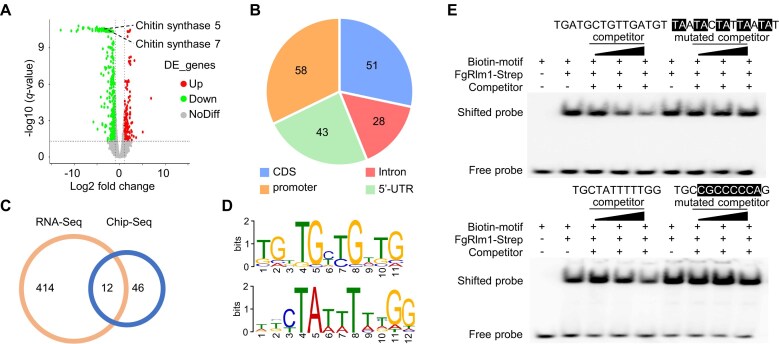
FgRlm1 binds to a 12- or 14-bp *cis*-element in the target promoters. (**A**) FgRlm1 is required for the upregulation of CWI-related genes including chitin synthase genes 5 and 7 in TBI condition. Scatter plot showing changes in gene expression as detected by RNA-seq in ΔFgRlm1 compared with the wild-type PH-1 cultured in TBI medium for 24 h. Up- and downregulated genes are highlighted in red and green, respectively. Expressed genes with fold change ≥2 and *P*-value ≤.05 were regarded as significantly different. Genes that did not meet these criteria are shown in gray (NoDiff). (**B**) Distributions of FgRlm1-binding peaks in coding sequence (CDS), intron, 5′-UTR, and promoter. ΔFgRlm1::FgRlm1-GFP grown in the TBI medium was used for ChIP with the anti-GFP antibody. (**C**) Overlap between the downregulated genes in ΔFgRlm1 and FgRlm1 binding genes. The left circle indicates genes downregulated in ΔFgRlm1 in RNA-seq compared with the wild type under TBI condition and the right circle indicates the genes whose promoters were bound by FgRlm1 in ChIP-seq. (**D**) The putative *cis*-element predicted by analyzing sequences of FgRlm1-binding promoters with the MEME program. (**E**) FgRlm1 binds two putative *cis*-elements in EMSA. The wild-type (WT) DNA fragment consisting of three repeats of the *cis*-element was labeled with biotin. Unlabeled WT or mutated DNA fragments were used as competitors at 50-, 100-, and 150-fold molar excess relative to the labeled probe. The mutated nucleotides are shaded in black.

To elucidate the mechanism of FgMgv1–FgRlm1 axis in regulating CWI, RNA-seq assays were performed for the ΔFgRlm1 mutant and the wild-type PH-1 under TBI conditions in three independent experiments. The results showed that a total of 426 genes were significantly downregulated (fold change ≥2, *P*-value ≤.05), with 51 of these genes exhibiting more than a four-fold reduction in expression (*P* ≤ .05) in ΔFgRlm1 as compared to those in PH-1 (Fig. 3A). Gene Ontology (GO) analysis indicated that FgRlm1 was involved in cell wall biosynthesis and remodeling pathways (Supplementary Fig. S9A). Moreover, genome-wide ChIP-seq assays showed that FgRlm1 was found to bind to the 1-kb promoter regions upstream of 58 ORFs consistently in two independent experiments (Fig. 3B). Among these targets, 12 genes were downregulated in ΔFgRlm1 (Supplementary Table S2), including chitin synthase genes (*FGSG_12039, FgCHS5*; *FGSG_11964, FgCHS7*), cell wall glucanase genes (*FGSG_03529*, *FGSG_13947*), and a gene encoding a cell wall anchoring protein (*FGSG_11149*) (Fig. 3C and Supplementary Fig. S9B).

To identify the FgRlm1 binding *cis*-elements in these target promoters, the MEME program was used and two *cis*-elements within the promoters of these 58 genes were predicted (Fig. 3D and Supplementary Table S3). Further analysis revealed that these *cis*-elements were present in the promoters at a significant percentage of 67.24% of the FgRlm1-binding peaks identified from the ChIP-seq assay (Supplementary Fig. S10 and Supplementary Table S3). Consistently, EMSA assays verified that FgRlm1 was able to bind to the identified *cis*-elements (Fig. 3E). Collectively, these data indicate that FgRlm1 regulates CWI under TBI conditions, primarily through the regulation of chitin synthase genes and other cell wall-related genes.

### FgRlm1 recruits FgMgv1 to the promoter regions of *FgCHS5* and *FgCHS7* and activates their transcription

**Figure 4.**
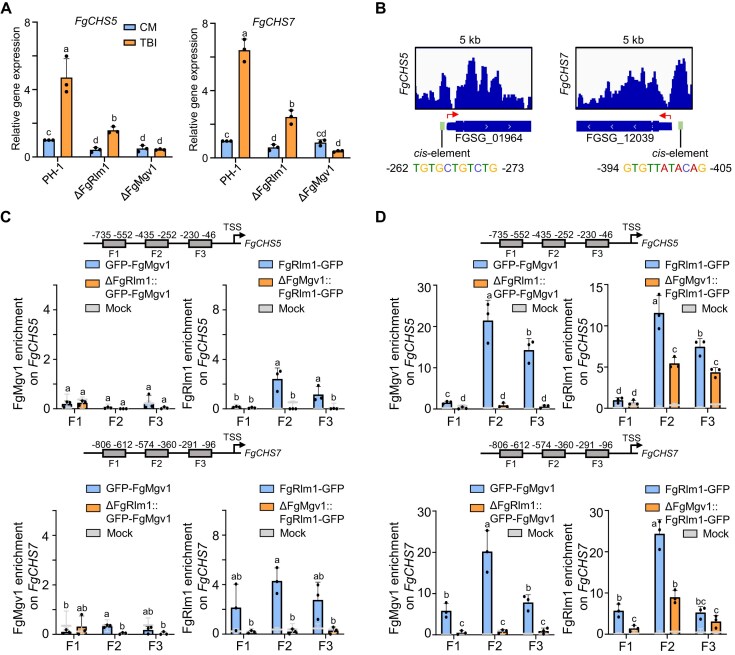
FgRlm1 recruits FgMgv1 to the promoter regions of *FgCHS5* and *FgCHS7* and activates their transcription. (**A**) *FgCHS5* and *FgCHS7* are downregulated in the ΔFgMgv1 and ΔFgRlm1 mutants compared to wild-type PH-1 under toxin-inducing conditions. RNA samples were extracted from fresh mycelia grown in CM medium for 16 h or in TBI medium for 24 h. Line bar in each column denotes standard deviation of three repeated experiments. Different letters indicate significant differences based on ANOVA followed by Fisher’s LSD test (*P*= .05). (**B**) Genome-browser view of FgRlm1 peaks and *cis*-element in the promoters of *FgCHS5* and *FgCHS7*. (**C**) Enrichment of FgRlm1 and FgMgv1 in the promoters of *FgCHS5* and *FgCHS7* under non-toxin inducing conditions. Fresh mycelia grown in CM medium for 16 h were used for ChIP–qPCR analysis. *X*-axis is the bp from transcription start site (TSS). Line bar in each column denotes the standard deviation of three repeated experiments. Different letters indicate significant differences based on ANOVA followed by Fisher’s LSD test (*P*= .05). (**D**) FgMgv1 is enriched in the promoters of *FgCHS5* and *FgCHS7* in a FgRlm1-dependent manner under toxin-inducing condition. Fresh mycelia grown in TBI medium for 24 h were used for ChIP–qPCR analysis.

Given that FgMgv1 lacks a DNA-binding domain, it was reasonable to hypothesize that FgRlm1 may recruit FgMgv1 to the promoters of target genes, including *FgCHS5* and *FgCHS7*. To test this hypothesis, the gene expression levels of *FgCHS5* and *FgCHS7* were examined in ΔFgMgv1 and ΔFgRlm1. The results revealed a two- to three-fold decrease in the expression levels of both genes in ΔFgRlm1 compared to the wild type (Fig. 4A). To further investigate enrichment of these proteins in target promoters, the GFP-labeled strains ΔFgMgv1::GFP-Mgv1, ΔFgMgv1::FgRlm1-GFP, ΔFgRlm1::GFP-Mgv1, and ΔFgRlm1::FgRlm1-GFP were constructed. In the ΔFgMgv1 mutant, the nuclear localization of FgRlm1 remained unchanged compared to that in the wild type, indicating that phosphorylation of FgRlm1 by FgMgv1 did not affect the subcellular localization of FgRlm1 (Supplementary Fig. S11). The enrichment of GFP-FgMgv1 and FgRlm1-GFP in the promoters of *FgCHS5* and *FgCHS7* containing different *cis*-elements of FgRlm1 was determined by ChIP–PCR (Fig. 4B–D). Under the CM condition, FgRlm1 but not FgMgv1 displays the enrichment at the promoter of *FgCHS5* and *FgCHS7* genes (Fig. 4C and Supplementary Fig. S12). However, under the TBI condition, both FgRlm1 and FgMgv1 were highly enriched in the promoter regions of *FgCHS5* and *FgCHS7* (Fig. 4D). Deletion of FgRlm1 blocked the binding ability of FgMgv1 to these promoter regions under TBI condition (Fig. 4D). Additionally, the enrichment of FgRlm1 in the promoters of these target genes was also decreased in ΔFgMgv1, but FgMgv1 does not affect the *in vitro* DNA-binding ability of FgRlm1 (Fig. 4C and D, and Supplementary Fig. S13), indicating that the enrichment of FgRlm1 to the target promoters is partially dependent on its phosphorylation by FgMgv1. These findings highlight the role of FgMgv1–FgRlm1 axis in regulating the expression of critical cell wall biosynthesis genes in *Fg*.

### Cooperation between SAGA and SWI/SNF complexes is required for cell wall integrity regulated by FgMgv1

**Figure 5.**
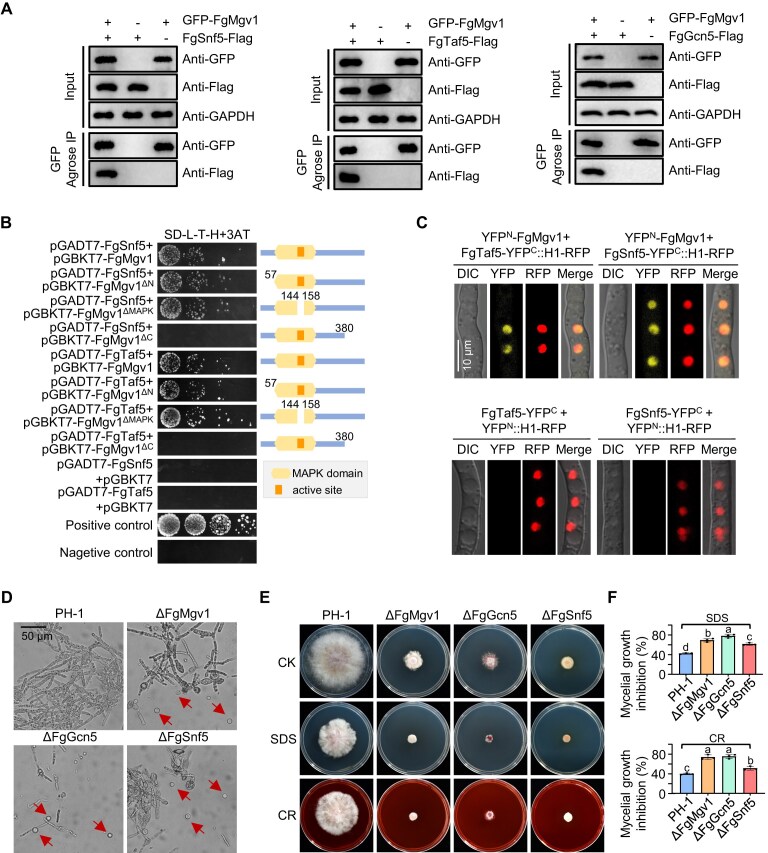
Cooperation of FgMgv1 with SAGA and SWI/SNF complexes is required for CWI. (**A**) FgMgv1 associates with FgSnf5, FgTaf5, and FgGcn5 in the co-IP assay. Protein samples were extracted from each strain incubated in TBI medium for 24 h. (**B**) FgMgv1 interacts with FgTaf5 and FgSnf5 via its C-terminus in Y2H assays. Yeast cells grew on SD-L-T-H + 3-AT. (**C**) FgMgv1 associates with FgTaf5 and FgSnf5 in nucleus in BiFC assays. The indicated strains were incubated in TBI medium for 24 h. (**D**) ΔFgMgv1, ΔFgGcn5, and ΔFgSnf5 are more sensitive to cell wall lytic enzymes. The wild-type PH-1, ΔFgMgv1, ΔFgGcn5, and ΔFgSnf5 grown in the TBI medium for 24 h were treated with cellulase, lysozyme, and driselase for 30 min at 30°C. (**E**, **F**) ΔFgMgv1, ΔFgGcn5, and ΔFgSnf5 display increased sensitivity to SDS and CR compared to the wild-type PH-1. Colony morphology (D) and mycelial inhibition (E) of each strain were examined after growth on MM medium with or without 0.02% SDS or 0.02% CR for 3 days.

Given that ΔFgMgv1 exhibits a more severe defect in hyphal growth than ΔFgRlm1, it is possible that FgMgv1 is associated with additional factors to maintain CWI as previously reported [15, 25, 26]. To further elucidate the mechanism of FgMgv1 in regulating CWI, Y2H screening against the *Fg* cDNA library was conducted using FgMgv1 as bait, identifying 62 proteins as the potential interacting protein, including FgTaf5 (a subunit shared by TFIID and of SAGA complexes) and FgSnf5 (a core subunit of SWI/SNF) (Supplementary Table S4). The interactions of FgMgv1 with FgTaf5 and FgSnf5 were further verified by co-IP in both TBI and CM condition (Fig. 5A and Supplementary Fig. S14A). Considering FgMgv1 does not interact with FgTaf8, which is TFIID-specific subunit with both co-IP and Y2H assays (Supplementary Fig. S15), we thus subsequently verified the interaction of FgMgv1 with FgGcn5, the key subunit of the SAGA complex, by co-IP (Fig. 5A). Moreover, Y2H assays demonstrated that FgMgv1 interacted with FgTaf5 and FgSnf5, but not with FgGcn5 (Fig. 5B and Supplementary Fig. S16). Moreover, the association of FgMgv1 with FgTaf5 and FgSnf5 in the nucleus was confirmed by BiFC under both TBI and CM conditions (Fig. 5C and Supplementary Fig. S14B). The kinase activation of Slt2 homologues is regulated by phosphorylation at threonine and tyrosine residues within the conserved TXY motif in the activation loop (T186 and Y188 in FgMgv1, corresponding to T190 and Y192 in Slt2 of *S. cerevisiae*, respectively) [46]. To investigate the effect of FgMgv1 kinase activation on its interaction with FgSnf5 and FgTaf5, we generated an inactivated FgMgv1 variant by introducing T186A and Y188F mutations within its activation loop of the MAPK. Noteworthy, FgSnf5 and FgTaf5 still can directly interact with FgMgv1 carrying mutations (T186A/Y188F) (Supplementary Fig. S17A–C), indicating that the interaction of FgMgv1 with FgTaf5 and FgSnf5 is independent on its kinase activity.

To investigate whether the SAGA complex and the SWI/SNF chromatin remodeling complex are involved in the cell wall stress response in *Fg*, we attempted to create gene knockout mutants for FgTaf5 and FgSnf5. Despite performing three independent transformation experiments and screening 500 transformants, we were unable to obtain a knockout mutant for FgTaf5, suggesting that FgTaf5 is essential and lethal when knocked out. Consequently, we constructed an FgGcn5 mutant to assess its sensitivity to cell wall-damaging agents and cell wall-degrading enzymes alongside the FgSnf5 mutant. Similar to ΔFgMgv1, ΔFgGcn5 and ΔFgSnf5 were digested into more protoplasts by the cell wall-degrading enzymes than the wild-type PH-1 (Fig. 5D). Consistently, ΔFgGcn5 and ΔFgSnf5 were markedly more sensitive to cell wall-damaging agents CR and SDS compared to the wild-type strain PH-1 (Fig. 5E and F). Furthermore, FgMgv1^T186A/Y188F^ cannot restore the sensitivity of ΔFgMgv1 to cell wall-damaging agents and lytic enzymes (Supplementary Fig. S17D–F). Overall, these results suggest that although FgMgv1 interacts with the SAGA and SWI/SNF complexes independently of its kinase activity, this activity is essential for maintaining CWI.

### FgMgv1 functions as a scaffold protein for SWI/SNF and SAGA complexes in targeted gene promoters

**Figure 6.**
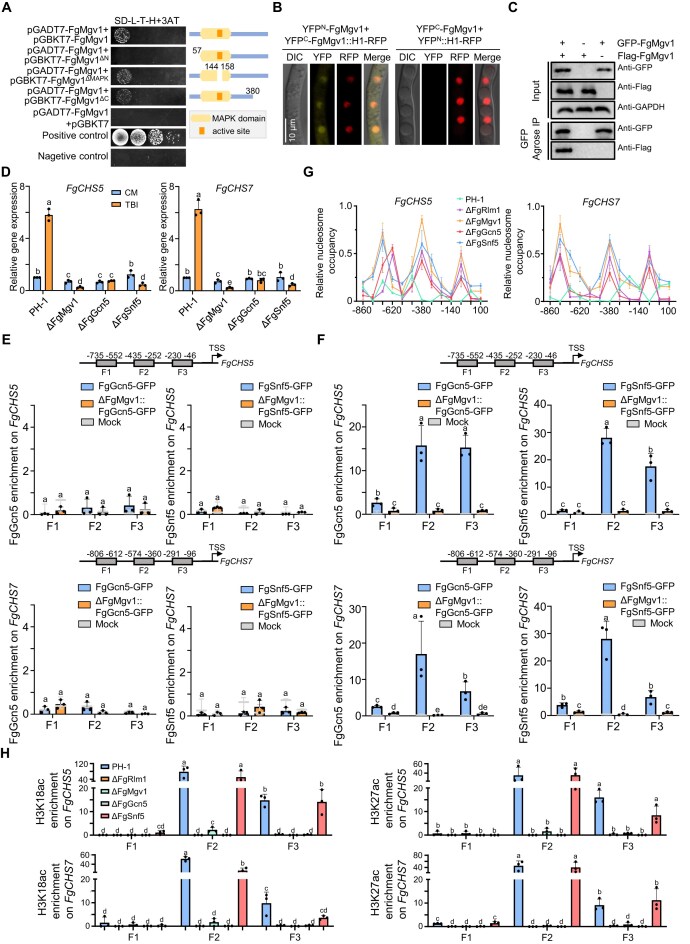
FgMgv1 functions as a scaffold protein for SWI/SNF and SAGA complexes in targeted gene promoters. (**A**) FgMgv1 forms a polymer via its N-terminus in Y2H assay. Interactions were determined by monitoring growth on SD-L-T-H + 3-AT of yeast cells bearing a pair of vectors as indicated. The pGBKT7-53 and pGADT7-T were used as positive control, and pGBKT7-Lam and pGADT7-T were used as negative control. (**B**) FgMgv1 forms a polymer in nucleus in BiFC assay. The strains containing YFP^N^-FgMgv1, YFP^C^-FgMgv1, and H1-RFP were incubated in TBI medium for 24 h and then images were taken under a confocal microscopy. (**C**) FgMgv1 interacts with itself in the co-IP assay. Protein samples were extracted from fresh mycelia of each strain incubated in TBI medium for 24 h, with protein loading amounts verified using the anti-GAPDH. (**D**) *FgCHS5* and *FgCHS7* are markedly downregulated in ΔFgSnf5 and ΔFgGcn5 compared to those in the wild-type PH-1 under TBI condition. RNA samples were extracted from fresh mycelia grown in CM medium for 16 h or in TBI medium for 24 h. Line bar in each column denotes standard deviation of three repeated experiments. Different letters indicate significant differences based on ANOVA, followed by Fisher’s LSD test (*P*= .05). (**E**) SAGA and SWI/SNF complexes are not enriched in the promoters of *FgCHS5* and *FgCHS7* under non-toxin inducing conditions. *X*-axis is the bp from TSS. Line bar in each column denotes standard deviation of three repeated experiments. Different letters indicate significant differences based on ANOVA followed by Fisher’s LSD test (*P*= .05). (**F**) FgGcn5 and FgSnf5 enrich on the promoters of *FgCHS5* and *FgCHS7* depending on FgMgv1 in TBI condition. (**G**) FgRlm1, FgMgv1, FgGcn5, and FgSnf5 mediate nucleosomal positioning rearrangement in promoters of *FgCHS5* and *FgCHS7*. The indicated strains grown in the TBI medium were used for MNase–qPCR assays. (**H**) FgMgv1, FgRlm1, FgGcn5, and FgSnf5 regulate the acetylation of H3K18 and H3K27 in the promoters of *FgCHS5* and *FgCHS7*. The indicated strains grown in the TBI medium were used for ChIP–qPCR analysis of H3K18 and H3K27 acetylation. Line bar in each column denotes standard deviation of three repeated experiments. Different letters indicate significant differences based on one-way ANOVA followed by Fisher’s LSD test (*P* = .05).

Since FgMgv1 interacts with FgRlm1, FgTaf5, and FgSnf5, the interactions of various truncated FgMgv1 with these proteins were investigated to figure out their interaction relationships. Interestingly, the interaction between FgMgv1 with FgTaf5 and FgSnf5 was dependent on its C-terminus, which contrasts with the FgMgv1 kinase domain-dependent interaction seen in interaction of FgMgv1 with FgRlm1 (Figs 2D and 5B). Unexpectedly, FgMgv1 may interact with itself in Y2H assay (Fig. 6A), BiFC (Fig. 6B), and co-IP (Fig. 6C), indicating that FgMgv1 may form polymer. Results of Y2H assay indicated that the N-terminus of FgMgv1 has no effect on its protein localization (Supplementary Fig. S18A), but is critical for interacting with itself (Fig. 6A) and for growth and resistance to cell wall lytic enzymes (Supplementary Fig. S18B and C).

Further, the expression of *FgCHS5* and *FgCHS7* in the wild type was upregulated to approximately six-fold under TBI compared to the CM condition, but not in ΔFgSnf5 and ΔFgGcn5, similar to that was seen in ΔFgMgv1 and ΔFgRlm1 (Fig. 6D). To further investigate how the FgMgv1–FgRlm1 axis regulates the epigenetic complexes, we detected the enrichment of FgGcn5 of SAGA complex and FgSnf5 of SWI/SNF complex on two target genes (*FgCHS5* and *FgCHS7*). The ChIP–qPCR results showed that FgGcn5 and FgSnf5 were significantly enriched in the promoter regions of target genes *FgCHS5* and *FgCHS7* under TBI but not CM condition, while the deletion of FgMgv1 resulted in a significant decrease in the enrichments of FgGcn5 and FgSnf5 (Fig. 6E and F). These results indicate that FgMgv1 functions as a scaffold protein for the transcription factor FgRlm1, and SWI/SNF and SAGA complexes in promoters of cell wall biosynthesis genes. Moreover, MNase–qPCR assays showed that nucleosome occupancy at the promoters of *FgCHS5* and *FgCHS7* genes in ΔFgRlm1, ΔFgMgv1, ΔFgGcn5, and ΔFgSnf5 were higher than that in PH-1 (Fig. 6G and Supplementary Fig. S19). Considering that FgGcn5 regulates the H3K18ac and H3K27ac in *Fg* [47, 48], we examined the enrichment of H3K18ac and H3K27ac at the promoters of *FgCHS5* and *FgCHS7*. The results showed that the levels of H3K18ac and H3K27ac were reduced in ΔFgRlm1, ΔFgMgv1, and ΔFgGcn5 but not in ΔFgSnf5 compared to that of wild-type PH-1 (Fig. 6H). These results indicate that the FgMgv1–FgRlm axis is necessary for recruiting SAGA and SWI/SNF complexes to synergistically regulate transcription of the genes involved in CWI.

### Regulation of fungal virulence and toxin synthesis by FgRlm1, FgMgv1, and SAGA and SWI/SNF complexes

**Figure 7.**
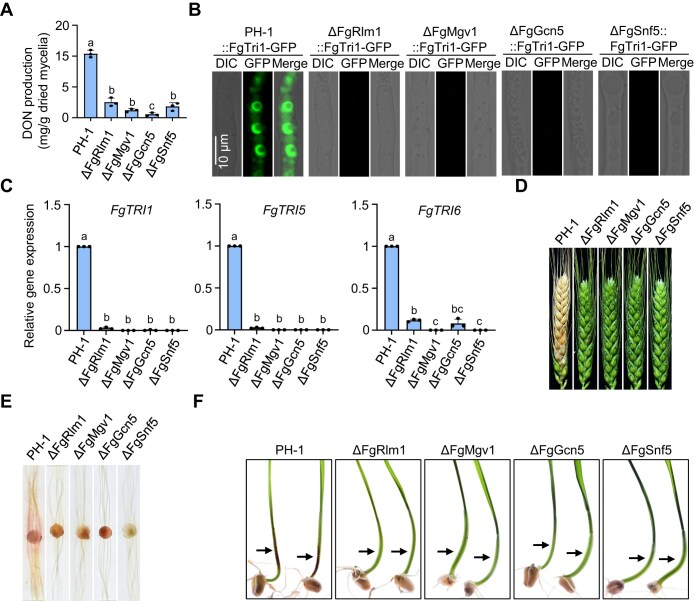
FgRlm1, FgMgv1, and SAGA and SWI/SNF complexes cooperatively regulate toxin biosynthesis and pathogenicity in *Fg*. (**A**) Deletion of FgRlm1, FgMgv1, FgSnf5, or FgGcn5 impairs DON production. The indicated strains were grown in TBI medium for 7 days before detection of DON production. Different letters indicate significant difference based on one-way ANOVA, followed by LSD test (*P* = .05). (**B**) Deletion of FgRlm1, FgMgv1, FgSnf5, or FgGcn5 impairs the formation of toxisome labeled by FgTri1-GFP. The strains were incubated in TBI medium for 36 h and then images were taken under a confocal microscope. (**C**) Deletion of FgRlm1, FgMgv1, FgSnf5, or FgGcn5 impairs the expression of *FgTRI1*, *FgTRI5*, and *FgTRI6*. RNA samples were extracted from fresh mycelia grown in TBI for 2 days. (**D**) FgRlm1, FgMgv1, FgSnf5, and FgGcn5 are required for the pathogenicity of *Fg* on wheat head. Fresh mycelia of each strain were inoculated on wheat heads for 15 days before the images were taken. (**E**) FgRlm1, FgMgv1, FgSnf5, and FgGcn5 are required for virulence of *Fg* on corn silk. Corn silks were examined after 4 days of inoculation with mycelial plugs of indicated strains. (**F**) FgRlm1, FgMgv1, FgSnf5, and FgGcn5 are required for virulence of *Fg* on wheat coleoptiles. Arrows indicate the corresponding coleoptiles lesion sites. Infected wheat coleoptiles were examined 4 days after inoculated with fresh mycelia of indicated strains.

Considering the involvement of FgRlm1, FgMgv1, and SAGA and SWI/SNF complexes in regulating the response of *Fg* to cell wall damage and the expression of genes involved in cell wall biosynthesis under TBI conditions, we investigated the production of DON in the deletion mutants ΔFgRlm1, ΔFgMgv1, ΔFgGcn5, and ΔFgSnf5. As shown in Fig. 7A, deletion of these genes significantly impaired DON production, which is in line with previous reports [6, 26, 47]. To further confirm this finding, we examined the formation of toxisome, the site for DON biosynthesis [49–51]. GFP was tagged to the ORF of *FgTRI1*, which encodes the key enzyme catalyzing the final step of DON biosynthesis. The resulting construct was introduced into ΔFgRlm1, ΔFgMgv1, ΔFgGcn5, ΔFgSnf5, and the wild-type PH-1. In the wild-type strain PH-1::FgTri1-GFP, but not in ΔFgMgv1::FgTri1-GFP, ΔFgGcn5::FgTri1-GFP, and ΔFgSnf5::FgTri1-GFP, FgTri1-GFP was highly induced and localized at spherical structures (DON toxisomes) after 48 h of incubation in TBI (Fig. 7B). Consistent with this result, the expression of *FgTRI1*, *FgTRI5*, and *FgTRI6* genes dramatically decreased in these mutants compared to that in the wild type (Fig. 7C). These results indicate that FgRlm1, FgMgv1, and the SAGA and SWI/SNF complexes play critical roles in DON biosynthesis.

Given the importance of DON as a virulence factor, we also investigated the regulatory roles of FgRlm1, FgMgv1, and SAGA and SWI/SNF complexes in the virulence of *Fg*. As shown in Fig. 7D, ΔFgRlm1, ΔFgMgv1, ΔFgGcn5, and ΔFgSnf5 mutants lost their ability to infect wheat heads after 15 days of inoculation, whereas the wild-type strain exhibited typical FHB symptoms. Consistently, these mutants displayed decreased virulence on corn stalks and wheat seedling sheaths (Fig. 7E and F). To determine whether the reduced virulence is solely caused by the lack of DON, we applied exogenous DON to individual wheat spikelet following inoculation. The results showed that DON partially restored the pathogenicity of these mutants (Supplementary Fig. S20). These findings suggest that FgRlm1, FgMgv1, and the SAGA and SWI/SNF complexes are essential for full virulence of *Fg*, which is partly attributable to impaired DON production.

## Discussion

**Figure 8.**
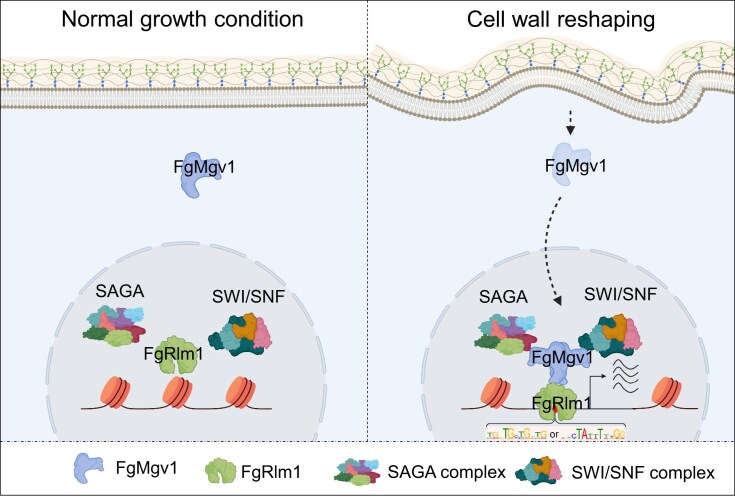
A proposed model for the orchestration of SAGA and SWI/SNF complexes by the FgMgv1–FgRlm1 axis in modulating cell wall integrity in *Fg*. Under TBI conditions, activated FgMgv1 translocates into the nucleus, where it interacts with the transcription factor FgRlm1, which recruits it to the promoters of cell wall biosynthesis genes. Subsequently, FgMgv1 forms a polymer and recruits the SAGA and SWI/SNF complexes, promoting target gene expression and maintaining cell wall integrity.

The fungal cell wall is crucial for viability, development, and pathogenicity, especially during host infection [52, 53]. Pathogenic fungi often encounter host-derived enzymes that can hydrolyze components of the fungal cell wall, serving as a defense mechanism against infection [54]. Therefore, maintaining CWI is vital for successful colonization. In this study, we found that the core regulator of the fungal CWI pathway, FgMgv1, is activated under TBI conditions and translocated into the nucleus to activate its downstream transcription factor, FgRlm1. Notably, FgRlm1 recruits FgMgv1 to the promoters of chitinase synthesis genes. There, FgMgv1 forms a polymer and acts as a scaffold protein, facilitating the recruitment of SAGA and SWI/SNF complexes to these promoters, and subsequently activating transcription (Fig. 8). This study elucidates a comprehensive regulatory framework in which FgMgv1 acts as a central scaffold protein, linking CWI signals with epigenetic modifications, thereby ensuring target gene transcription in response to environmental stimuli as the previous studies [15, 25, 29].

MAPKs are ubiquitous and highly conserved kinase in all eukaryotes, which serve as key signal transducers via protein phosphorylation/dephosphorylation cycles [21, 55]. The MAPK, Slt2, can activate GAL1–LacZ transcription in response to cell wall stress when fused to the Gal4 DNA-binding domain (DBD) [15, 25]. Similarly, Slt2, fused with the Rlm1 DBD artificially, is also able to target to the promoter of CWI-dependent gene and induce gene transcription in the absence of Rlm1 [15, 25, 29]. These studies imply the role of MAPK in directly regulating gene transcription, besides as a protein kinase. In the study, we found that the FgMgv1 forming a polymer serves as a scaffold protein to coordinate the SAGA and SWI/SNF complexes. Unlike its interaction with FgRlm1, which occurs through the MAPK domain, the association with the SAGA and SWI/SNF complexes is mediated by the C-terminus of FgMgv1. In addition, the kinase-inactivated mutant of FgMgv1 (T186A/Y188F) still interacts with SAGA and SWI/SNF complexes, which highlights the function of FgMgv1 beyond its kinase activity.

The SAGA complex is an evolutionarily conserved transcriptional coactivator that regulates gene expression through its histone acetyltransferase and deubiquitylase activities, recognition of specific histone modifications, and interactions with transcription factors [5, 16, 56, 57]. For example, the transcription factor Gcn4 recruits the SAGA complex to the promoters of its target genes for transcriptional activation [58]. Unlike SAGA, SWI/SNF complexes are not specifically associated with either active or repressive transcriptional states and their effect on transcription appears to be context-dependent, potentially due to the combinational assembly of different subunits [59–61]. In addition, given the low abundance of the SWI/SNF complex within cells and its non-sequence-specific interaction with nucleosome DNA, the recruiters, especially transcription factor, may guide these complexes to specific genomic regions [5, 7]. For instance, our previous studies showed that the transcription factors FgAreB and FgSR may recruit SWI/SNF for nitrosative stress response and ergosterol biosynthesis, respectively [6, 62]. In yeast, Rlm1 likely recruits SAGA and SWI/SNF complexes for CWI-responsive genes expression in an MAPK Slt2-dependent manner [15, 25, 29]. However, the study shows that FgMgv1 directly recruits the SWI/SNF and SAGA complexes (Fig. 5A–C). Supportively, FgMgv1, FgGcn5, and FgSnf5 mutants show more severe growth than the FgRlm1 mutant (Figs 2B and 5E), indicating that the recruitment by FgMgv1 is not limited to the target regions of FgRlm1. In addition, FgMgv1 may also have other downstream targets that are independent of SAGA and SWI/SNF complexes. In line with this, approximately one-sixth of the genes of the fungal genome are required to generate the cell wall, which is also beyond the ability of Rlm1 [30]. It would be thus interesting to identify other downstream outputs of FgMgv1 that are involved in CWI in *Fg* and other pathogenic fungi.

While the basic physiological blueprint of the fungal cell wall in terms of its biophysical design is conserved, the fungal cell wall is adaptable and can accommodate the requirements of various fungal lifestyles [30]. These properties enable fungi to undergo morphogenesis and survive a range of environmental stresses. For instance, infection cushions of *Fg* were observed during infection on wheat [63, 64]. Interestingly, DON biosynthesis is highly induced in the infection cushions [65] (Supplementary Fig. S5), although DON is not required for the initial penetration of wheat tissues [50, 51]. In addition, we observed that during wheat infection, the mycelia of *Fg* were more resistant to digestion and generated fewer protoplasts compared to mycelia grown in a 2% sucrose solution (Fig. 1D). These observations suggest that the CWI pathway is activated during fungal infection and DON biosynthesis. However, the relationship between DON biosynthesis and activation of the CWI pathway remains unclear. This study found that the activated FgMgv1–FgRlm1 axis is crucial for DON biosynthesis, suggesting that this axis not only contributes to resistance against plant-degrading enzymes to maintain CWI but also promotes an intracellular environment favorable for DON production during infection of *Fg*.

## Supplementary Material

gkaf653_Supplemental_Files

## Data Availability

Raw reads of ChIP-seq and RNA-seq generated in this study have been deposited in the National Genomics Data Center (https://ngdc.cncb.ac.cn/) under project accession code PRJCA029352 (https://ngdc.cncb.ac.cn/bioproject/browse/PRJCA029352) as project number and PRJCA029346 (https://ngdc.cncb.ac.cn/bioproject/browse/PRJCA029346).
