# Supplementary material for: The Mgv1–Rlm1 axis orchestrates SAGA and SWI/SNF complexes at target promoters
Source: Nucleic Acids Res. 2025 Jul 12;53(13):gkaf653. doi: 10.1093/nar/gkaf653 (PMC12255304; doi:10.1093/nar/gkaf653)
Supplement: gkaf653_Supplemental_Files [file gkaf653_supplemental_files.zip › Supplementary Figs with Figure legend.pdf]

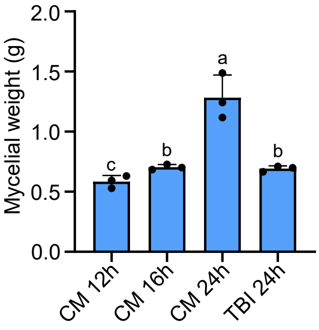

Supplementary Fig. 1 Wild-type strain growth in CM medium for 16 h and in TBI medium for 24 h have similar biomass .

Wild-type strain PH-1 with same quantities were growth in CM medium for 12 h, 16 h and 24 h or in TBI medium for 24 h. The mycelium is filtered, dried, and then weighed.

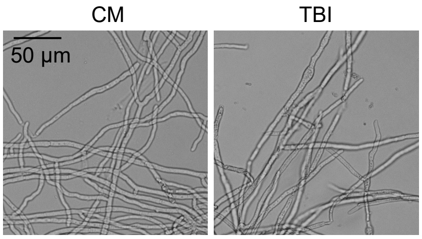

Supplementary Fig. 2 Negative control of mycelia without cell wall lytic enzymes.
Wild type PH-1 grown in the CM or TBI medium without cellulase, lysozyme and driselase. Bar = 50 µm.

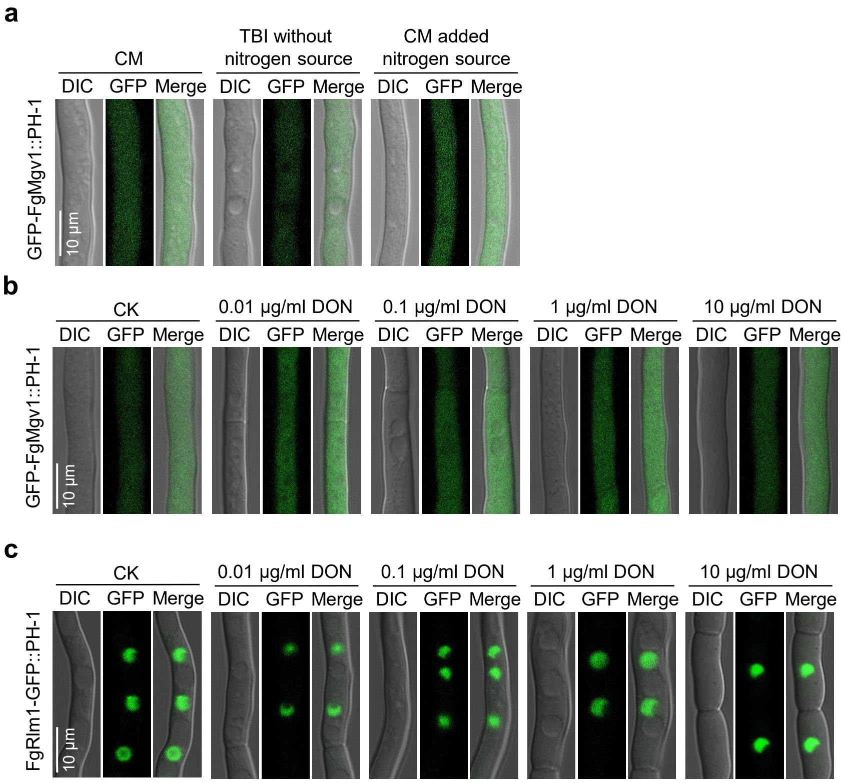

Supplementary Fig. 3 CWI pathway is not activated by exogenous DON or TBI medium lacking a nitrogen source.

(a) CWI pathway is not activated by TBI medium lacking a nitrogen source. Strains was incubated in CM medium, TBI medium without nitrogen source or CM medium added nitrogen source. Bar = 10 μm.

(b) Exogenous DON does not cause FgMgv1 to enter the nucleus. Strains was incubated in CM medium for 16 h, then different concentrations of DON were added externally and treated for an additional 6 h. Bar = 10 μm.

(c) Exogenous DON does not alter the localization of FgRlm1. Strains was incubated in CM medium for 16 h, then different concentrations of DON were added externally and treated for an additional 6 h. Bar = 10 μm.

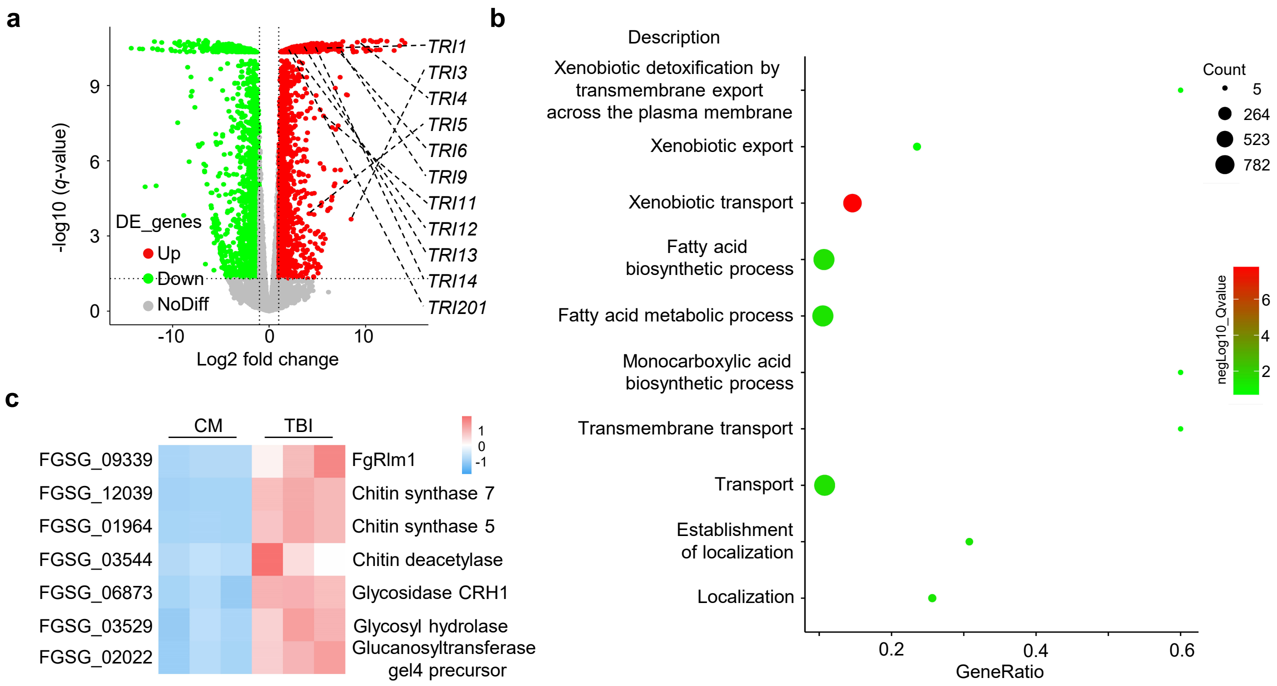

Supplementary Fig. 4 Cell wall biosynthesis genes are up-regulated in wild-type strain under TBI conditions.

(a) *TRIs* gene are up-regulated under TBI condition. Scatter plot showing comparisons in expression levels in RNA-seq assays among wild-type strain cultured in CM medium for 16 h and in TBI medium for 24 h. Up- and down-regulated genes are highlighted in red and green, respectively. The remaining genes are shown in grey. Expressed genes with fold-change ≥ 2 and *P*-value ≤ 0.05 were regarded as significantly different.

(b) Gene Ontology (GO) analysis pathway enrichment analysis for differentially expressed genes between CM and TBI conditions. Differentially expressed genes are defined by *P* ≤ 0.05 and log 2 fold change ≥ 2 or ≤ -2.

(c) Cell wall related genes are induced under TBI conditions. The heatmap was constructed to visualize the fold changes in expression of these genes, based on RNA-seq data from a wild-type strain cultured in CM medium for 16 h and in TBI medium for 24 h.

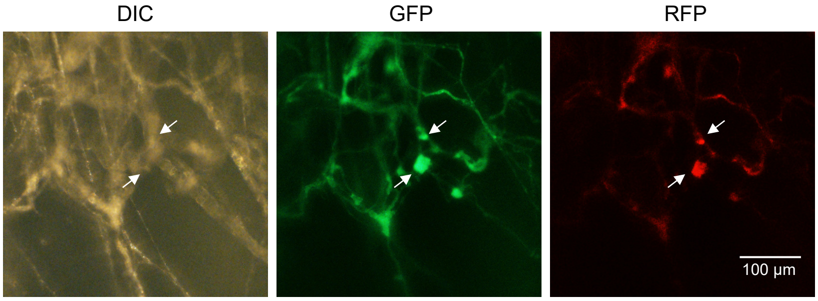

Supplementary Fig. 5 Infection condition activates the CWI pathway in *F. graminearum*

FgRlm1-GFP and FgTri5-RFP are induced in the infection cushion structure (indicated by arrows) when the strain PH-1::FgRlm1-GFP::FgTri5-RFP was inoculated on wheat leaf for 2 d.

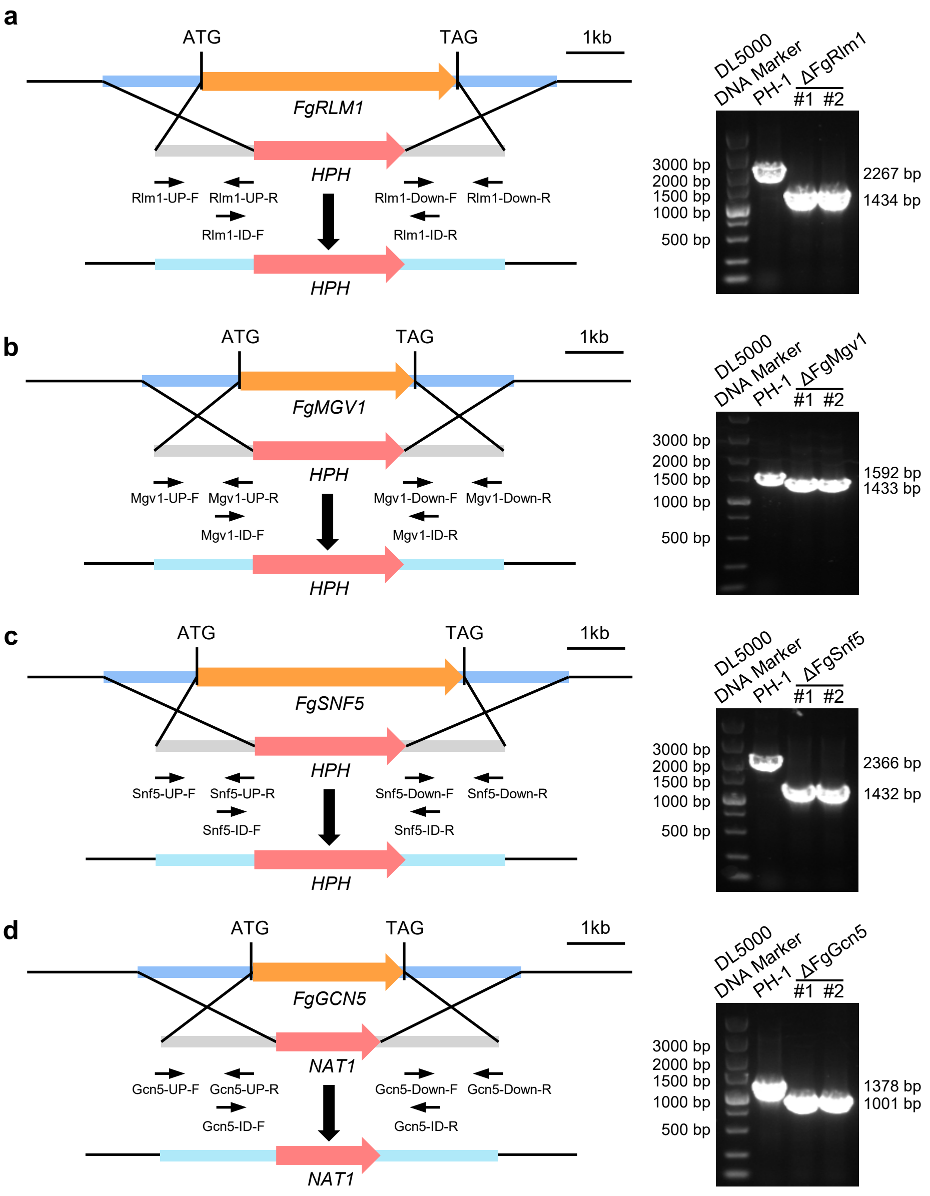

Supplementary Fig. 6 Generation of *FgRLM1*, *FgMGV1*, *FgSNF*, and *FgGCN5* deletion mutant. (a-d) Schematic representation of the *FgRLM1* (a), *FgMGV1* (b), *FgSNF5* (c), and *FgGCN5* (d) disruption strategy and PCR assays for identification of corresponding genes deletion mutant. Binding positions of PCR primers (arrows) used for the construction of gene deletion mutant are illustrated (left panel). Correct deletion was confirmed by PCR assay (right panel) using the primer pairs ID-F/ ID-R indicated in left panel.

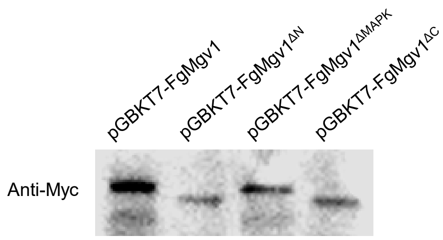

Supplementary Fig. 7 Truncated proteins of FgMgv1 used in Y2H assay are stable.

Protein samples were extracted from Y2H Gold with different vectors.

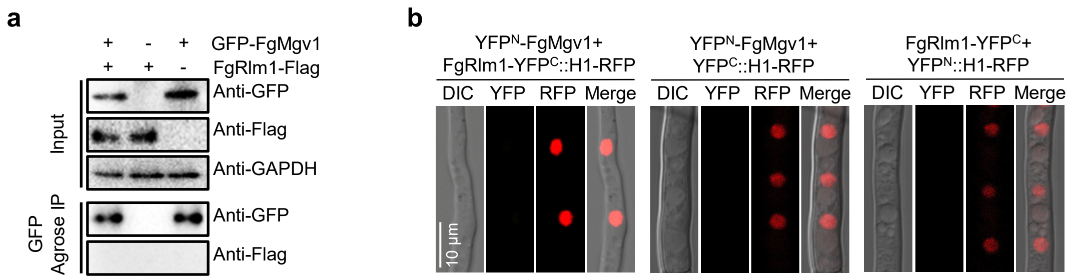

Supplementary Fig. 8 FgMgv1 does not interact with FgRlm1 under non-toxin inducing conditions.

(a) FgMgv1 does not interact with FgRlm1 under non-toxin-inducing conditions in the Co-IP assay. Protein samples were extracted from fresh mycelia of each strain incubated in CM medium for 16 hours, with protein loading amounts verified using the anti-GAPDH.

(b) FgMgv1 does not associated with FgRlm1 under non-toxin-inducing conditions in BiFC assay. These strains was incubated in CM medium for 16 h. Bar = 10 μm.

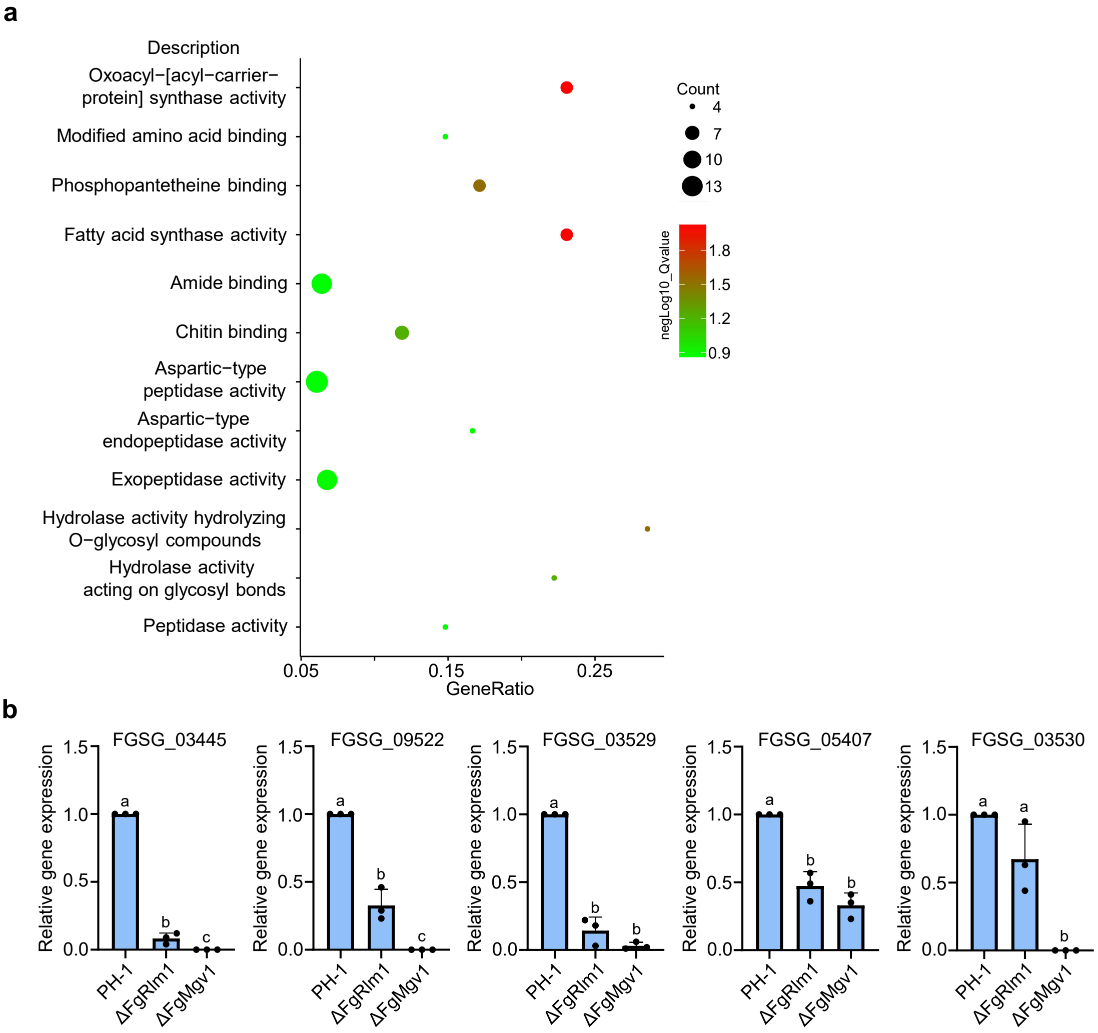

Supplementary Fig. 9 RNA-Seq of ΔFgRlm1 under toxin-inducing conditions.

(a) Gene Ontology (GO) analysis pathway enrichment analysis for differentially expressed genes between ΔFgRlm1 and wild-type PH-1 under TBI condition. Differentially expressed genes are defined by $P \leq 0.05$ and log 2 fold change ≥ 2 or ≤ - 2.

(b) Deletion of FgMgv1 or FgRlm1 impairs the expression of cell wall related genes under TBI conditions. RNA samples were extracted from fresh mycelia of indicated strains grown in TBI medium for 24 h. Line bar in each column denotes standard deviation of three repeated experiments. Different letters indicate significant differences based on ANOVA followed by Fisher's LSD test ($P = 0.05$).

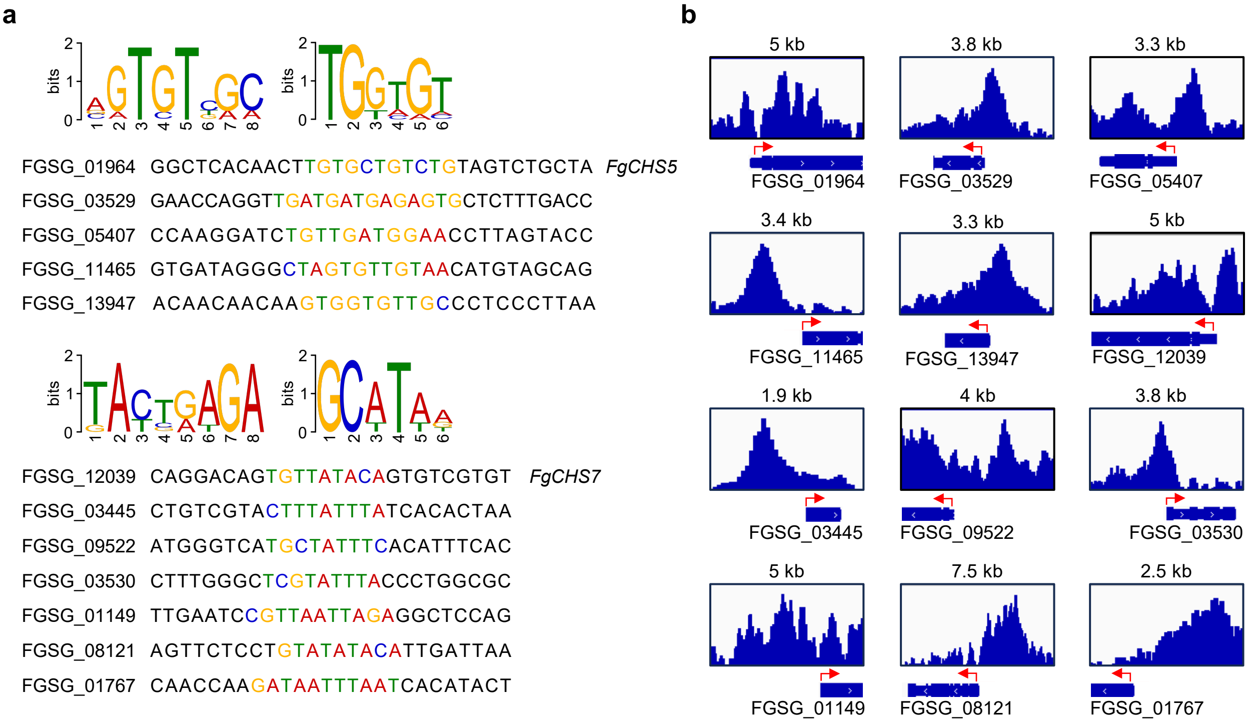

Supplementary Fig. 10 Genome-wide identification of FgRlm1-binding sites.

(a) The putative *cis*-elements present in the prompters of 12 CWI-related genes.

(b) Genome-browser view of FgRlm1 ChIP-seq peaks at the promoter of CWI-related genes.

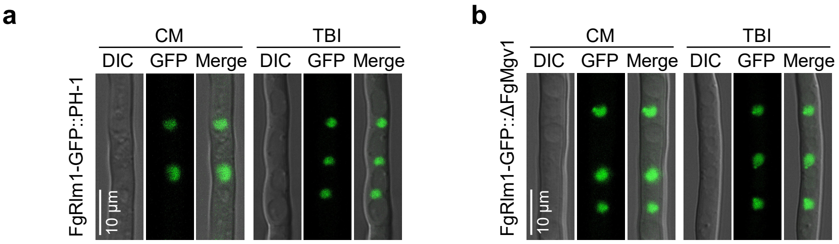

Supplementary Fig. 11 Protein localization of FgRlm1.

(a) The nuclear localization of FgRlm1 remains unchanged under non-toxin inducing conditions and toxin inducing conditions. Strains was incubated in CM medium for 16 h or in TBI medium for 24 h. Bar = 10 μm.

(b) Knockout of FgMgv1 does not alter the protein localization of FgRlm1 under non-toxin inducing conditions and toxin inducing conditions. Strains was incubated in CM medium for 16 h or in TBI medium for 24 h. Bar = 10 μm.

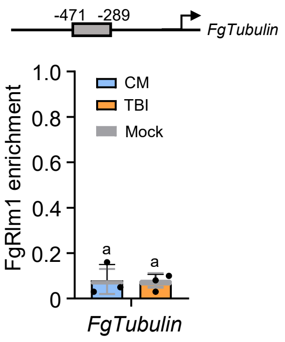

Supplementary Fig. 12 FgRlm1 is not enriched into the promoter of *FgTubulin*.

Line bar in each column denotes standard deviation of three repeated experiments. Different letters indicate significant differences based on ANOVA followed by Fisher's LSD test (P = 0.05).

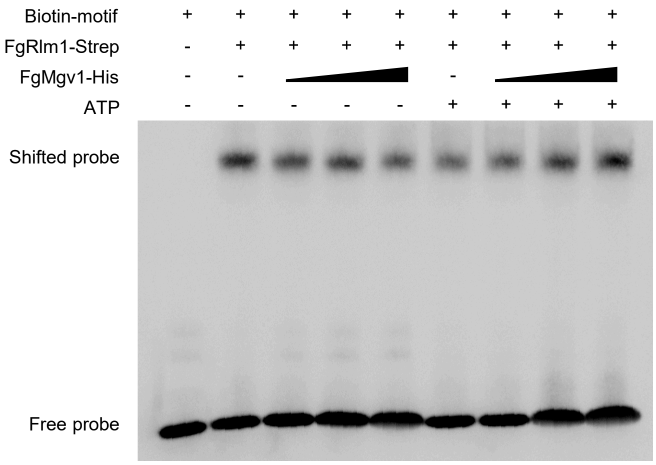

Supplementary Fig. 13 FgMgv1 does not alter the DNA-binding ability of FgRlm1 *in vitro*. Verification of the binding of FgRlm1 with the *cis*-element by electrophoretic mobility shift assay (EMSA). DNA fragments, FgRlm1 and a concentration of 1, 3 or 6-folds of FgMgv1-His with or without 10 μM ATP (Adenosine Triphosphate) were mixed. Mixtures were incubated in 37 ° C for 30 min before detection.

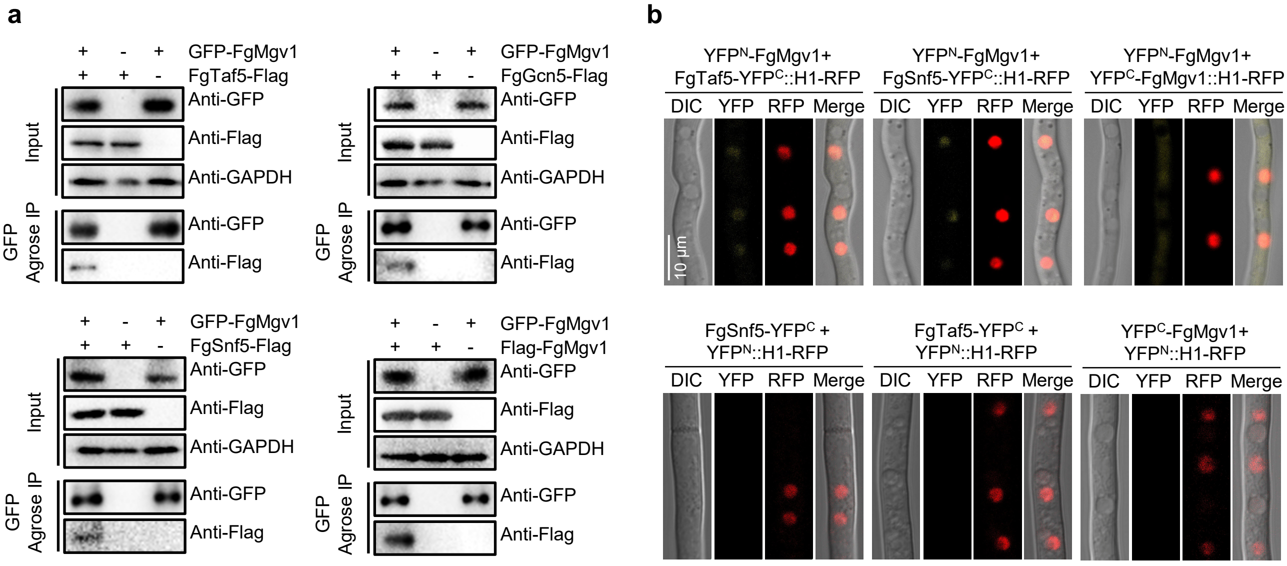

Supplementary Fig. 14 FgMgv1 interacts with subunits of SAGA and SWI/SNF complex and FgMgv1 itself under non-toxin inducing conditions.

(a) FgMgv1 associates with subunits of SAGA and SWI/SNF complex and FgMgv1 under non-toxin-inducing conditions in the Co-IP assay. These strains was incubated in CM medium for 16 h. Bar = 10 μm.

(b) FgMgv1 interacts with subunits of SAGA and SWI/SNF complex and FgMgv1 under non-toxin-inducing conditions in BiFC assay. Protein samples were extracted from fresh mycelia of each strain incubated in CM medium for 16 h, with protein loading amounts verified using the anti-GAPDH.

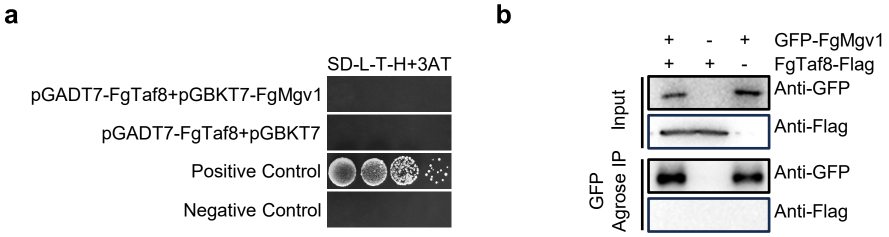

Supplementary Fig. 15 FgMgv1 does not interact with TFIID complex under toxin-inducing conditions.

(a) FgMgv1 does not associat with the subunit of TFIID, FgTaf8 in Y2H assay. Interactions were determined by monitoring the growth on synthetic defined (SD) medium lacking leucine (L), tryptophan (T), and histidine (H), but supplemented with 3-aminotriazole (3AT) (SD-L-T-H+3AT) of yeast cells bearing a pair of vectors as indicated. pGBKT7-53 and pGADT7-T were used as positive control, and pGBKT7-Lam and pGADT7-T were used as negative control.

(b) FgMgv1 does not interact with FgTaf8 under toxin-inducing conditions in the Co-IP assay. Protein samples were extracted from fresh mycelia of each strain incubated in TBI medium for 24 h.

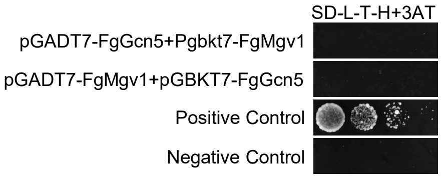

Supplementary Fig. 16 FgMgv1 does not interact with the core subunit of SAGA complex, FgGcn5 in Y2H assay.

Interactions were determined by monitoring the growth on synthetic defined (SD) medium lacking leucine (L), tryptophan (T), and histidine (H), but supplemented with 3-aminotriazole (3AT) (SD-L-T-H+3AT) of yeast cells bearing a pair of vectors as indicated. pGBKT7-53 and pGADT7-T were used as positive control, and pGBKT7-Lam and pGADT7-T were used as negative control.

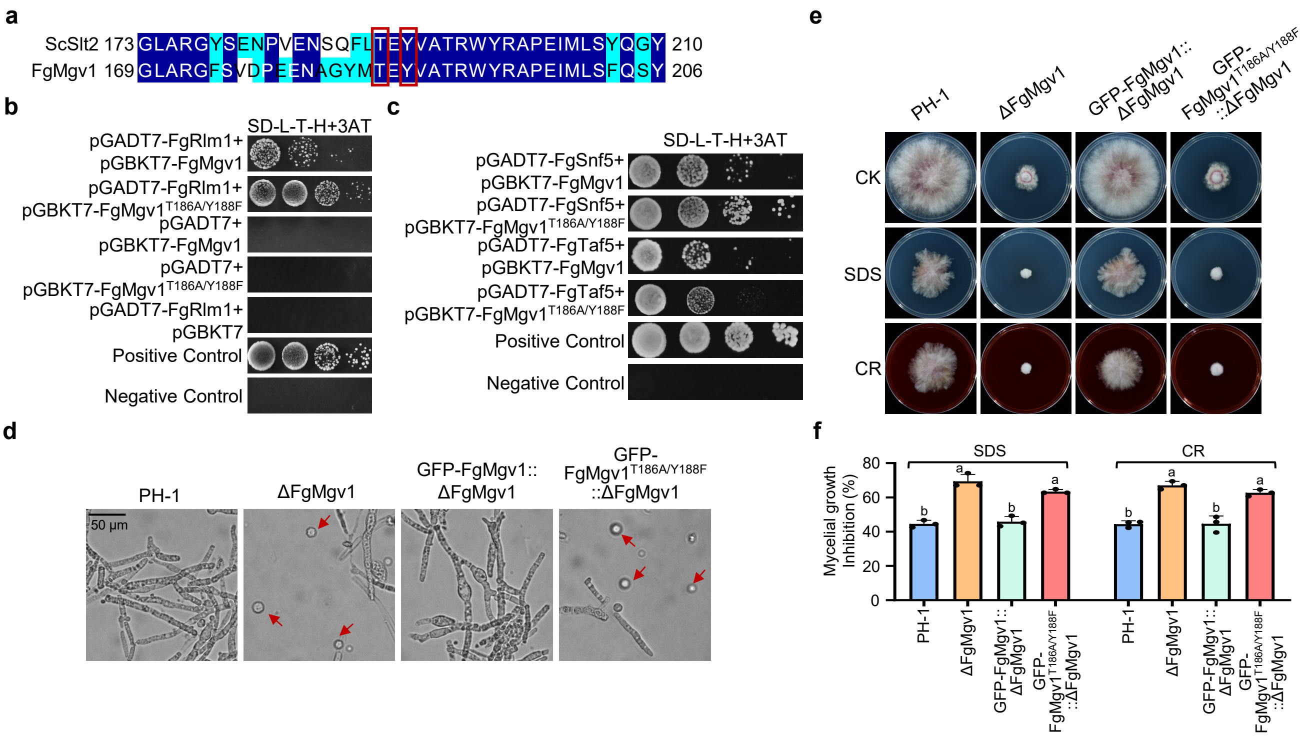

Supplementary Fig. 17 FgSnf5 and FgTaf5, FgRlm1 interacts with the inactive kinase FgMgv1.

(a) The alignment of FgMgv1 and Slt2 in kinase activation loop region.

(b) FgMgv1 interacts with FgRlm1 in kinase independent manner. Interactions were determined by monitoring the growth on synthetic defined (SD) medium lacking leucine (L), tryptophan (T), and histidine (H), but supplemented with 3-aminotriazole (3AT) (SD-L-T-H+3AT) of yeast cells bearing a pair of vectors as indicated. pGBKT7-53 and pGADT7-T were used as positive control, and pGBKT7-Lam and pGADT7-T were used as negative control.

(c) FgMgv1 interacts with FgTaf5 and FgSnf5 in kinase independent manner.

(d) FgMgv1 kinase inactive mutant cannot restore the sensitivity of ΔFgMgv1 to cell wall lytic enzymes. The indicated strains grown in the TBI medium for 24 h were treated with cellulase, lysozyme, and driselase for 30 min at 30° C. Bar = 50 μm.

(e, f) FgMgv1 kinase inactive mutant cannot restore the sensitivity of ΔFgMgv1 to cell damage agents. Colony morphology (e) and mycelial inhibition (f) was examined after growth on MM medium with or without 0.02 % SDS or 0.02 % CR for 3 days.

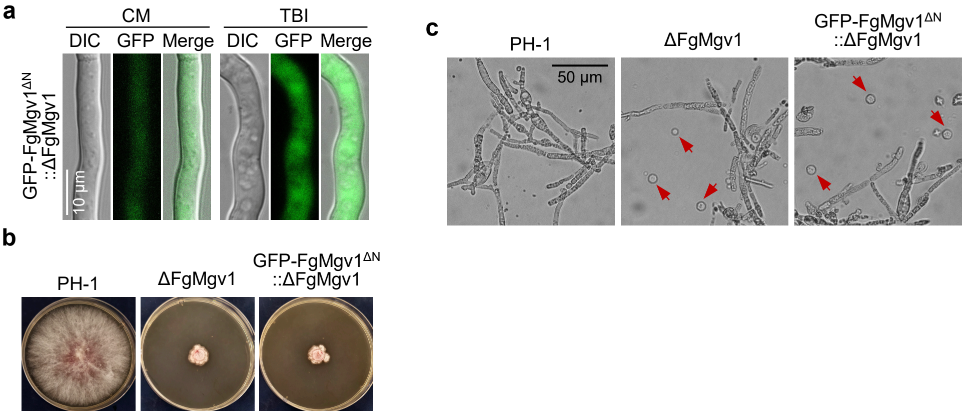

Supplementary Fig. 18 Protein oligomerization of FgMgv1 is essential for cell wall integrity.

(a) Defect of oligomerization do not alter FgMgv1 localization. Strains was incubated in CM medium for 16 h or in TBI medium for 24 h. Bar = 10 μm.

(b) Comparisons in colony morphology among the wild-type PH-1, ΔFgMgv1 and GFP-FgMgv1Δ^{N} ::ΔFgMgv1 growing on potato dextrose agar medium.

(c) GFP-FgMgv1Δ^{N}::ΔFgMgv1 cannot restore the sensitivity of ΔFgMgv1 to cell wall lytic enzymes. The indicated strains grown in the TBI medium for 24 h were treated with cellulase, lysozyme, and driselase for 30 min at 30° C.

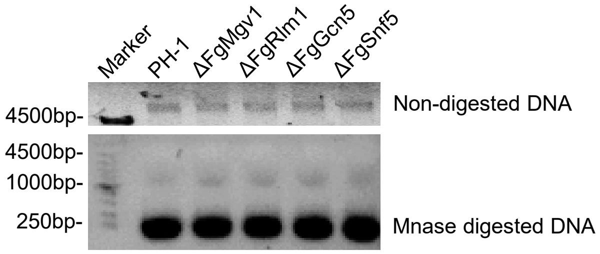

Supplementary Fig. 19 Different DNAs achieved similar MNase digestion level.

The gels showed similar MNase digestion levels of above different DNA samples after 8 min digestion.

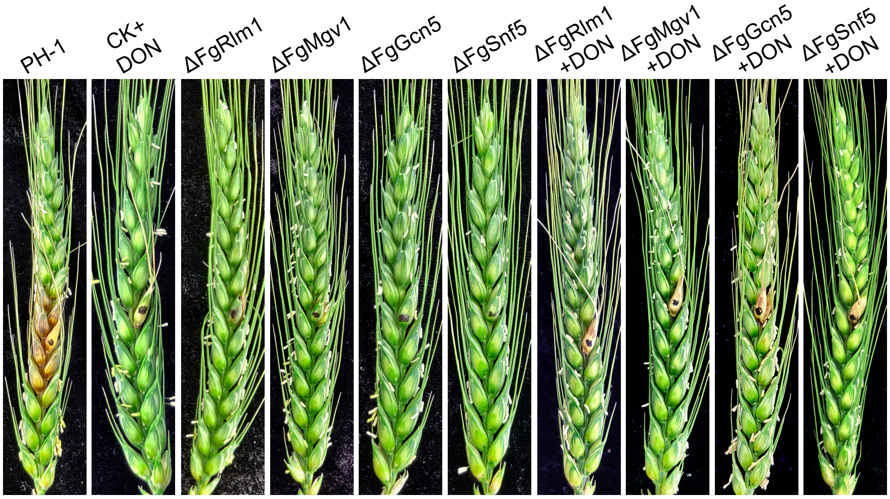

Supplementary Fig. 20 Exogenous DON partially restores the loss of pathogenicity in *FgMgv1*, *FgRlm1*, *FgGcn5* and *FgSnf5* mutants.

Exogenous DON partially restores the loss of pathogenicity in *FgMgv1*, *FgRlm1*, *FgGcn5* and *FgSnf5* mutants. Fresh mycelia of each strain with or without 10 μg/ml DON were inoculated on single wheat spike. Images were taken after 15 days.
